# Cyclic-AMP regulates postnatal development of neural and behavioral responses to NaCl in rats

**DOI:** 10.1371/journal.pone.0171335

**Published:** 2017-02-13

**Authors:** Jie Qian, Shobha Mummalaneni, Tam-Hao T. Phan, Gerard L. Heck, John A. DeSimone, David West, Sunila Mahavadi, Deanna Hojati, Karnam S. Murthy, Mee-Ra Rhyu, Andrew I. Spielman, Mehmet Hakan Özdener, Vijay Lyall

**Affiliations:** 1 Departments of Physiology and Biophysics, Virginia Commonwealth University, Richmond, Virginia, United States of America; 2 Korea Food Research Institute, Bundang-gu, Sungnam-si, Gyeonggi-do, Korea; 3 NYU College of Dentistry, New York, NY, United States of America; 4 Monell Chemical Senses Center, Philadelphia, Pennsylvania, United States of America; The University of Tokyo, JAPAN

## Abstract

During postnatal development rats demonstrate an age-dependent increase in NaCl chorda tympani (CT) responses and the number of functional apical amiloride-sensitive epithelial Na^+^ channels (ENaCs) in salt sensing fungiform (FF) taste receptor cells (TRCs). Currently, the intracellular signals that regulate the postnatal development of salt taste have not been identified. We investigated the effect of cAMP, a downstream signal for arginine vasopressin (AVP) action, on the postnatal development of NaCl responses in 19–23 day old rats. ENaC-dependent NaCl CT responses were monitored after lingual application of 8-chlorophenylthio-cAMP (8-CPT-cAMP) under open-circuit conditions and under ±60 mV lingual voltage clamp. Behavioral responses were tested using 2 bottle/24h NaCl preference tests. The effect of [deamino-Cys^1^, D-Arg^8^]-vasopressin (dDAVP, a specific V2R agonist) was investigated on ENaC subunit trafficking in rat FF TRCs and on cAMP generation in cultured adult human FF taste cells (HBO cells). Our results show that in 19–23 day old rats, the ENaC-dependent maximum NaCl CT response was a saturating sigmoidal function of 8-CPT-cAMP concentration. 8-CPT-cAMP increased the voltage-sensitivity of the NaCl CT response and the apical Na^+^ response conductance. Intravenous injections of dDAVP increased ENaC expression and γ-ENaC trafficking from cytosolic compartment to the apical compartment in rat FF TRCs. In HBO cells dDAVP increased intracellular cAMP and cAMP increased trafficking of γ- and δ-ENaC from cytosolic compartment to the apical compartment 10 min post-cAMP treatment. Control 19–23 day old rats were indifferent to NaCl, but showed clear preference for appetitive NaCl concentrations after 8-CPT-cAMP treatment. Relative to adult rats, 14 day old rats demonstrated significantly less V2R antibody binding in circumvallate TRCs. We conclude that an age-dependent increase in V2R expression produces an AVP-induced incremental increase in cAMP that modulates the postnatal increase in TRC ENaC and the neural and behavioral responses to NaCl.

## Introduction

Changes in gustatory sensitivity to salty taste occur during postnatal development in mammals [1]. Human infants at birth are indifferent to salt or reject it. By 4–6 months of age, they show a preference for isotonic and hypertonic saline solutions over water [2, 3]. In rats, during 7 to 50 days postnatal, responses of peripheral and central taste neurons increase several fold to stimulation of the anterior tongue with NaCl [4–8]. In the rat neonate chorda tympani (CT) taste nerve the Na^+^-selective fibers accounted for the increase in response with age [6]. Action potentials in these fibers were blocked by amiloride [9, 10]. The increase in rat NaCl CT response with age was also blocked by amiloride, indicating that the taste transducer being added with age is a Na^+^-specific ion channel [11]. In fungiform (FF) taste receptor cells (TRCs), the Na^+^ specific salt taste receptor is the amiloride- and benzamil (Bz)-sensitive epithelial Na^+^ channel (ENaC) [12–22]. In FF taste buds functional ENaCs are expressed in Type I cells [23]. TRC ENaC is composed of αβγ subunits in rodents and/or δβγ subunits in humans. The δβγ-ENaC demonstrates 26–30 fold higher IC_50_ values for amiloride and Bz relative to αβγ-ENaC [18, 24, 25]. δ-ENaC gene is absent in rats and mice [18, 26].

Rodents and other herbivorous mammals, rely on TRC ENaC to find Na^+^-salts in the environment [27, 28]. While they are still nursing, the animals receive dietary Na^+^ in mother’s milk. During this period functional TRC ENaC is not critical for survival. TRC ENaC must, therefore, be fully functional by the time the young animals begin to forage for food independently. Although an increment in ENaC expression and activity is seen in both reabsorptive epithelia and non-epithelial tissues in a development-dependent manner [26], a delay in ENaC activation does not occur in other tissues where such a delay would put the animal’s survival at risk [29]. Currently, little is known about the role of hormones and their downstream intracellular signaling effectors in the postnatal development of mammalian TRC ENaC. In addition to aldosterone [15], insulin [30], angiotensin II [31], ghrelin and ghrelin O-acyltransferase [32, 33], arginine vasopressin (AVP) and 3',5'-cyclic adenosine monophosphate (cAMP) have been shown to modulate TRC ENaC activity in adult rats and mice [21, 34–36]. In this paper, we investigated the role of cAMP in the postnatal development of neural and behavioral responses in the developing rats. Cyclic AMP is a downstream intracellular effector of G protein-coupled receptors that couple to G_s_ alpha subunit (Gα_s_). AVP binds to arginine vasopressin receptor-2 (V2R) coupled to Gα_s_. Gα_s_ stimulates adenylyl cyclase and increases cAMP followed by the activation of downstream effectors resulting in the trafficking of aquaporin-2 (AQP-2) and ENaC to the apical cell membrane [37–46]. TRCs contain several aquaporin isoforms, including AQP-2 [47]. The expression of adenylyl cyclase 4, 6, and 8 has been demonstrated in TRCs [48]. The effects of AVP are independent of aldosterone [49].

We investigated the effect of cAMP on neural and behavioral responses in 19–23 day old rats. In FF TRCs an incremental increase in cAMP was induced by topical lingual application of varying concentrations (5–20 mM) of a membrane permeable form of cAMP, 8-chlorophenylthio-cAMP (8-CPT-cAMP) for 10–15 min. Whole nerve CT responses to NaCl (0.1, 0.3 and 0.5 M) were monitored under open-circuit conditions (i.e. under zero voltage clamp) and under ±60 mV lingual voltage clamp before and after 8-CPT-cAMP treatment. The NaCl CT responses were monitored in the absence and presence of cetylpyridinium chloride (CPC), a specific blocker of the amiloride-insensitive component of the NaCl CT response or benzamil (Bz), a specific blocker of ENaC [50]. The tonic NaCl CT responses were normalized to 0.3 or 0.5 M NH_4_Cl and the data were fitted to an apical ENaC kinetic model [51]. Standard 2 bottle preference tests were used to evaluate fluid intake and NaCl preference in 19–23 day old and 60+ day old (adult) rats before and after 8-CPT-cAMP treatment. The effect of [deamino-Cys^1^, D-Arg^8^]-vasopressin (dDAVP, a potent and specific V2R agonist) was investigated on ENaC subunit trafficking in rat FF TRCs and on cAMP generation in cultured human adult FF taste cells (HBO cells) [52] using immunofluorescence and radioimmunoassay, respectively. For 8-CPT-cAMP experiments, the membrane permeable form of cGMP, 8-(4-Chlorophenylthio)-guanosine 3′,5′-cyclic monophosphate (8-CPT-cGMP) was used as a control.

The results presented here demonstrate that in 19–23 day old rats, an incremental increase in TRC cAMP mimics the postnatal age-dependent increase in the NaCl CT response and behavior. These changes are accompanied by a concomitant increase in the number of functional apical ENaC density in FF TRCs. Both dDAVP and cAMP increased ENaC expression and ENaC subunit trafficking from cytosolic compartment to the apical compartment in FF TRCs. Relative to adult rats, 14 day old rats demonstrated significantly less V2R antibody binding in circumvallate TRCs. We conclude that an age-dependent increase in V2R expression produces an AVP-induced incremental increase in cAMP that modulates the postnatal increase in TRC ENaC and the neural and behavioral responses to NaCl.

## Materials and methods

### Agonists and antagonists

8-(4-Chlorophenylthio)-adenosine-3',5'-cyclic monophosphate (8-CPT-cAMP), sodium salt, 8-(4-Chlorophenylthio)-guanosine 3′,5′-cyclic monophosphate (8-CPT-cGMP) sodium salt, benzamil (Bz), cetylpyridinium chloride (CPC), 3-Isobutyl-1-methylxanthine (IBMX), forskolin, arginine vasopressin (AVP), [deamino-Cys^1^, D-Arg^8^]-vasopressin (dDAVP), trichloroacetic acid, sodium pentobarbital, and isoflurane were obtained from Sigma-Aldrich.

### Antibodies and q-PCR primers

Western blots were used to detect the presence of α- and γ-ENaC subunits in rat TRCs. Antiserum against rat α- or γ-ENaC subunit was generated by immunizing rabbits with specific α- or γ-rENaC subunit peptides described earlier [53] using a standard immunization protocol (Pierce Biotechnology, Rockford, IL). V2R ((P-20)-R sc-1800-R) rabbit polyclonal antibody was obtained from Santa Cruz Biotechnology. The primary antibody for δ-hENaC (rabbit polyclonal anti-SCNN1D antibody (aa411-460) LS-C119717) was obtained from LifeSpan Biosciences and was used only on HBO cells. The secondary antibody, donkey anti-rabbit IgG-CFL 488 was obtained from Santa Cruz Biotechnology. Q-PCR primers for α- and γ-rENaC were obtained from Invitrogen. The primary antibodies for β-actin and HRP-conjugated secondary antibody were obtained from Santa Cruz Biotechnology. ECL Western Blotting Substrate was obtained from Life Technologies.

### Studies using rats

#### CT taste nerve recordings

The animals were housed in the Virginia Commonwealth University (VCU) animal facility in accordance with institutional guidelines. All *in vivo* animal protocols were approved by the Institutional Animal Care and Use Committee of VCU. Developing (19–23 day old) and adult (60+ day old) Sprague-Dawley rats were anesthetized by intraperitoneal injection of pentobarbital (60 mg/Kg) and supplemental pentobarbital (20 mg/Kg) was administered as necessary to maintain surgical anesthesia. The animal’s corneal reflex and toe-pinch reflex were used to monitor the depth of surgical anesthesia. Body temperatures were maintained at 36–37^°^C with an isothermal pad (Braintree Scientific, Braintree MA). The left CT nerve was exposed laterally as it exited the tympanic bulla and placed onto a 32G platinum/iridium wire electrode. An indifferent electrode was placed in nearby tissue. Stimulus solutions maintained at room temperature were injected into a Lucite chamber affixed by vacuum to a 28 mm^2^ patch of anterior dorsal lingual surface. The chamber was fitted with separate Ag-AgCl electrodes for measurement of current and potential and served as inputs to a voltage-current clamp amplifier that permitted the recording of CT responses with the chemically stimulated receptive field under zero current-clamp or voltage-clamp. The clamp-voltages were referenced to the mucosal side of the tongue [54]. For stimulation or rinsing, 3-ml aliquots were injected at a rate of 1 ml/s into the perfusion chamber. Typically, stimulus solutions remained on the tongue for 1–2 min. Control stimuli consisting of 0.3 or 0.5 M NH_4_Cl applied at the beginning and at the end of experiment were used to assess preparation stability. The preparation was considered stable only if the difference between the magnitude of the control stimuli at the beginning and at the end of the experiment was less than 10% [21, 55]. Neural responses were differentially amplified with a custom built, optically-coupled isolation amplifier. For display, responses were filtered using a band pass filter with cutoff frequencies 40 Hz-3 KHz and fed to an oscilloscope. Responses were then full-wave rectified and integrated with a time constant of 1s. Integrated neural responses were recorded on a chart recorder and also captured on disk using Labview software and analyzed off-line [21, 55]. The CT responses were recorded while the rat tongue was stimulated with a rinse solution (10 mM KCl) and then with stimulating solutions containing NaCl (0.1, 0.3 and 0.5 M) under current-clamp or voltage-clamp conditions. Benzamil (Bz; 5 μM) and cetylpyridinium chloride (CPC; 2 mM) were added to the NaCl solutions to block the ENaC-dependent and the Bz-insensitive component of the NaCl CT response, respectively [50].

CT responses were measured before and after topical lingual application of 8-CPT-cAMP, a membrane permeable form of cAMP. 8-CPT-cAMP (5–20 mM) was dissolved in H_2_O and was topically applied to the tongue for 10–15 min to enhance ENaC activity in TRCs. A membrane permeable form of cGMP, 8-CPT-cGMP was used as a control. In some experiments, adult rats were injected intravenously with AVP (1 nano mole/Kg BW) or dDAVP (0.1 nano moles/Kg BW) dissolved in normal saline and CT responses were monitored before and 15 min post-injection. The data were fitted to an apical ENaC kinetic model [51]. The equations used for data analysis are described in the Appendix.

#### Behavioral studies

Behavioral studies were performed in 19–27 day old and 60+ day old (adult) rats using standard two bottle/24h NaCl preference tests. Rats (21 day old) were anesthetized with isoflurane (2.5%) using the Surgivet/Anesco ISOTEC4 apparatus [56]. A small piece of a filter paper containing 50 μl of either H_2_O (Control) or 50 μl of 20 mM 8-CPT-cAMP dissolved in H_2_O or 50 μl of 20 mM 8-CPT-cGMP dissolved in H_2_O was placed on the anterior tongue for 20 min. Following this, the rat tongues were thoroughly washed with H_2_O to remove any remaining applied cAMP or cGMP. Individually 19 to 27 day old rats consume a small volume of fluid/day that is difficult to measure accurately. Accordingly, 3–5 rat pups were placed in each plastic cage and were given a choice between 2 bottles, one containing H_2_O and the other containing NaCl (0.035, 0.075 or 0.150 M). Their combined 24h fluid intake was monitored daily for the next 6 days. The volumes consumed for each solution were converted to g of fluid consumed by taking into account the density of each solution. The preference ratio for a taste stimulus was calculated as the g of the test solution consumed/24h/g mean body weight (BW) of rats divided by the total fluid intake (g of H_2_O/24h/g mean BW + g of the test solution/24h/g mean BW). The bottles containing H_2_O and the NaCl solution were switched from left to right at each trial.

Behavioral studies were performed in 3 groups of 14–16 adult rats/group. Before the start of the experiment rats were maintained on two bottles containing H_2_O for 7 days. After 7 days, the mean preference ratio for two bottles containing water was 0.50 ± 0.03. Rats were anesthetized with isoflurane and their tongues were treated with H_2_O or 20 mM 8-CPT-cAMP or 20 mM 8-CPT-cGMP for 20 min as described above. Rats were then placed individually in plastic cages and were given a choice between 2 bottles, one containing H_2_O and the other varying concentrations (0–0.5 M) of NaCl. Their 24h fluid intake was monitored daily for the next 6 days as described before [57]. The bottles containing H_2_O and the NaCl solution were switched from left to right at each trial. The data are expressed as means ± SEM of the number of rats in each group, and statistical significance was determined using Student's t-test for unpaired values.

#### Western-blot

To prepare protein samples, fifteen 14–19 day old rats and ten 60+ day old rats were used. The circumvallate (CV) papillae were isolated as described earlier [58] and pooled together and were lysed in modified RIPA buffer (50 mM Tris-Cl; pH 7.4, 1% NP-40, 150 mM NaCl, 1 mM EDTA, 1 mM PMSF, 1 μg/ml each of aprotinin and leupeptin, and 1 mM Na_3_VO_4_). The Western blot was performed with SDS-PAGE electro-blotting system (Bio-Rad). Briefly, 40 μg total protein samples were resolved by 10% SDS-PAGE and transferred to nitrocellulose membranes (Cat: 162–0094, Bio-Rad). Membranes were immune-blotted with rabbit antiserum containing α-rENaC antibody and a primary β-actin antibody followed by HRP-conjugated secondary antibodies. Reactions were visualized by ECL Western Blotting Substrate. Beta-actin was used as a protein loading control.

#### Quantitative(Q)-PCR

Rat CV papillae were collected from 15 day old and 60+ day old rats. Total RNA was isolated by using RNAqueous Micro-kit (cat AM 1931, life technologies). Q-PCR was used to measure RNA transcripts of α-rENaC and γ-rENaC. Primers used for Q-PCR were obtained from Invitrogen: α-rENaC (Rn00580652_m1) and γ-rENaC (Rn00566891_m1). Results were calculated using the 2^−ΔΔCt^ method based on GAPDH (Rn01775763_g1) amplification.

#### Immunofluorescence studies

Fifteen day old rats were injected intravenously with 1 nano moles of dDAVP/Kg BW, 3 times, 2h apart, and were sacrificed 24h later. The control rats were injected with saline. Animals were perfused with 4% paraformaldehyde/1×PBS for 5–10 min under anesthetic (isoflurane). The tongues were excised and fixed in 4% paraformaldehyde/1 × PBS for 2h at 4°C, and dehydrated in 40% sucrose/1×PBS overnight at 4°C before embedding in O.C.T. Compound (Andwin Scientific, Cat 14-373-65). Sections (8 μm thick) were prepared using a CM3050S cryostat (Leica Microsystems) and applied on pre-coated microscope slides (Fisher Scientific, Cat 12-550-15). The sections were dried at room temperature for 20 min and immediately used for immunofluorescence. After washing with 1X PBS for 5 min and blocking with 3% donkey serum for 1h at room temperature, sections were stained with rabbit antiserum containing γ-rENaC antibody (1:100 dilution in 3% donkey serum) at 4°C for overnight. After washing, sections were incubated with fluorescent-conjugated secondary antibodies for 1h at room temperature. Nuclei were visualized with 1 μg/ml of 4',6-diamidino-2-phenylindole (DAPI). Images were acquired with a 40X oil immersion objective on a Zeiss LSM 700 confocal laser scanning microscope and processed using Photoshop software (Adobe System). In another set of naïve adult rats (90+ day old) and 14 day old rat pups, V2R antibody binding was investigated in CV taste buds. Rat kidney was used as a positive control.

### Studies with HBO cells

#### Cell culture

HBO cells were cultured as described earlier [52].

#### Detection of ENaC subunit mRNA by PCR

Total RNA from HBO cells was purified using TRIzol reagent (cat# 15596018, Thermo Fisher Scientific, MA, USA) and reverse transcription was performed using High-Capacity cDNA Reverse Transcription Kit (cat# 4368814, Thermo Fisher Scientific, MA, USA). RT-PCR for the detection of α-, β-, γ-, and δ-ENaC subunits was carried out by using MyTaq red mix (Bioline, Luckenwalde, Germany). Briefly, 2 μg total RNA was mixed with 2× Reverse Transcription Master Mix to total 20 μl per reaction. Reverse transcription were performed at 25°C X 10 min, then 37°C X 120 min, followed by 85°C X 5 sec and cooled to 4°C. Subsequently, 200 ng total cDNA was used as template, 35 cycles of PCR amplification were performed (initial denaturation at 95°C for 1 min, denaturation at 95°C for 15 sec, annealing for 15 sec at 59–60°C, and extension for 10 sec at 72°C). RT-PCR products were subjected to electrophoresis on a 1% agarose gel to determine the expression of ENaC subunits and other taste receptors. The primers used to detect the presence of mRNAs in HBO cells for the α-, β-, γ-, and δ-ENaC subunits were synthesized by Thermo Fisher Scientific. The primer pairs used to detect the mRNA of the ENaC subunits are listed in Table 1.

**Table 1:** Primers used for RT-PCR.

Gene	NCBI accession number	Primer	Length
α-ENaC	NM_001038.5		
Forward		TCGAGTTCCACCGCTCCTA	166 bp
Reverse		GCCAGTACATCATGCCAAAGG	
β-ENaC	NM_000336.2		
Forward		CAGGACCTACTTGAGCTGGGA	170 bp
Reverse		CCAGGATTCTCTCCAGGACAG	
γ-ENaC	NM_001039.3		
Forward		CCGACCATTAAAGAGCTGATGC	120 bp
Reverse		AGTCAGTGTGAACCCGATCCA	
δ-ENaC	NM_001130413		
Forward		CCATCAGCATCCGAGAGGAC	186 bp
Reverse		GAGGGTGGAGGTAGTAGCCA	

#### Detection of δ-ENaC protein by western blot

HBO cells (2 x 10^6^) were lysed in 200 μl modified RIPA buffer (50 mM Tris-Cl; pH 7.4, 1% NP-40, 150 mM NaCl, 1 mM EDTA, 1 mM PMSF, 1 μg/ml each of aprotinin and leupeptin, and 1 mM Na_3_VO_4_). Samples containing 30 μg total protein were used for the experiment. The Western blot was performed with SDS-PAGE electro-blotting system (Bio-Rad) as described above for CV taste bud cells. HEK 293-cells were used as positive control. HEK cells were obtained from (American Type Culture Collection, Manassas, VA) and grown in a complete 293 SFMII growth medium (Thermo Fisher Scientific) supplemented with 4 mM L-glutamine. Cells were maintained at 37°C in 5% CO_2_.

### cAMP assay

Cyclic-AMP levels were measured in the presence of the phosphodiesterase blocker IBMX [59] as described earlier [60]. HBO cells (2 x 10^6^ cells/ml) were incubated for 10 min in the presence of 100 μM IBMX in the absence and presence of 0.01 μM dDAVP, 0.1 μM dDAVP or 10 μM forskolin. The reaction was terminated with 1 ml of 6% ice-cold trichloroacetic acid, and cAMP was extracted by freeze thawing. The acid was removed by ether extraction, and cAMP was measured in triplicates by radioimmunoassay using 10 μl aliquots of reconstituted samples. The results were expressed in picomoles per mg protein.

### Immunofluorescence

HBO cells (1 x 10^4^ cells/well) were plated into 8-wells chamber slides and treated with 10 μM 8-CPT-cAMP or 10 μM 8-CPT-cGMP or 10 nM dDAVP for 10 min. Following that HBO cells were fixed with 4% PFA for 10 min at 4°C. The procedure of immunofluorescence staining was same as described for taste bud cells above. After washing with 1X PBS for 5 min and blocking with 3% donkey serum for 1h at room temperature, cells were stained with rabbit antiserum containing γ-rENaC antibody (1:400 dilution in 3% donkey serum) or rabbit polyclonal δ-ENaC antibody (1:100 dilution in 3% donkey serum) at 4°C for overnight. After washing, cells were incubated with fluorescent-conjugated secondary antibodies for 1h at room temperature. Nuclei were visualized with 1 μg/ml of 4',6-diamidino-2-phenylindole (DAPI). Images were acquired with a 40X oil immersion objective on a Zeiss LSM 700 confocal laser scanning microscope and processed using Photoshop software (Adobe System). To quantitate the number of δ-ENaC positive HBO cells, the confocal images were divided into 4 regions of interest (ROIs). In each ROI, the total number of cells were counted as DAPI labelled nuclei and the δ-ENaC positive cells as green fluorescence labelled cells.

## Results

### Separation of ENaC-dependent and ENaC-independent NaCl CT response

**Fig 1:**
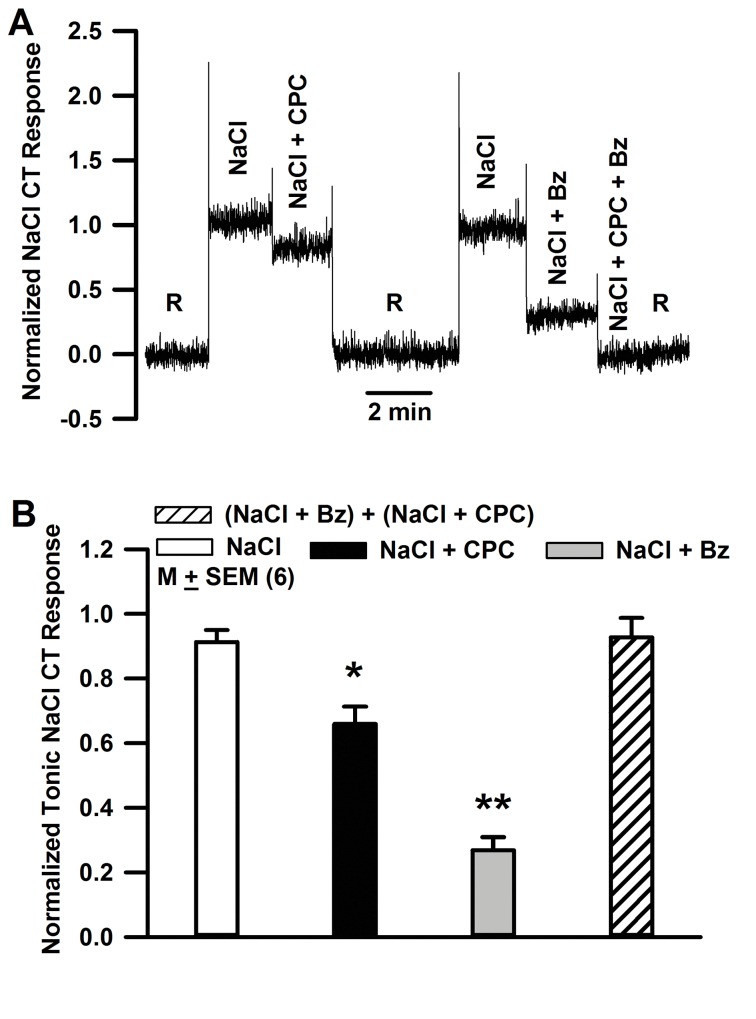
Effect of CPC and Bz on the NaCl CT response in adult rats. **(A)** Shows a representative NaCl CT response in an adult (60+ day old) rat. CT responses were monitored under open-circuit conditions while the tongue was first rinsed with a rinse solution R (10 mM KCl) and then with 0.3 M NaCl. The CT responses were also monitored with NaCl stimulating solutions containing 2 mM CPC or 5 μM Bz or 2 mM CPC + 5 μM Bz. **(B)** Shows the mean normalized tonic CT responses to NaCl, NaCl + CPC, NaCl + Bz and the added response ((NaCl + CPC) + (NaCl + Bz)) from 6 rats. The data were normalized to 0.5 M NH_4_Cl tonic response. *p = 0.0031; **p = 0.0001. The mean tonic CT responses to NaCl + CPC and NaCl + Bz were also significantly different with p = 0.0002.

In adult rats, the NaCl CT response is composed of two components (Fig 1). One component is blocked by CPC and the second component is blocked by Bz. Bz is a specific blocker of ENaC. Thus, the Bz-sensitive response represents the component of the NaCl CT response that arises due to Na^+^ influx through apical ENaCs in salt sensing FF TRCs. The Bz-insensitive response is blocked by CPC. It represents the ENaC-independent component of NaCl CT response. This component most likely arises due to apical Na^+^ influx through non-selective cation channel(s) in TRCs that are sensitive to CPC [50]. To investigate specifically changes in ENaC-dependent NaCl CT responses during postnatal development, NaCl CT responses in 19–23 day old rats were monitored in the presence of CPC. CPC completely eliminates the ENaC-independent component of the NaCl CT response.

### Effect of 8-CPT-cAMP and 8-CPT-cGMP on NaCl CT responses in 19–23 day old rats

**Fig 2:**
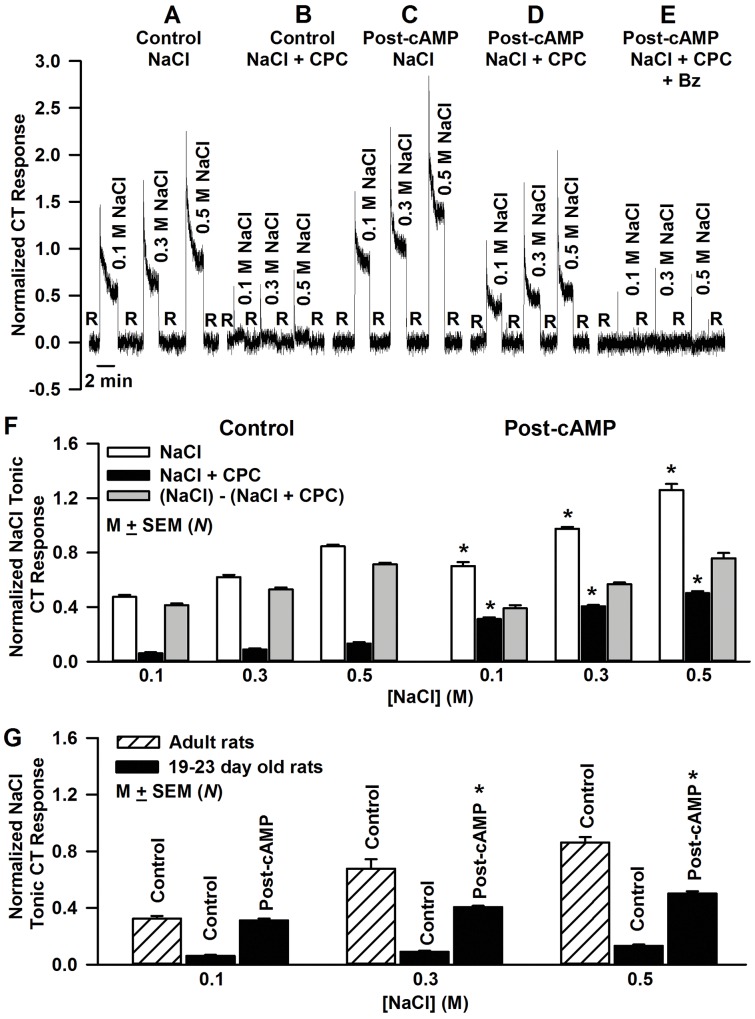
Effect of 8-CPT-cAMP on the NaCl CT responses in 19–23 day old rats. The figure shows a representative response in a 21 day old rat, in which the CT responses were monitored under open-circuit conditions while the tongue was first rinsed with a rinse solution R (10 mM KCl) and then with 0.1, 0.3 and 0.5 M NaCl under controls conditions **(A)** and in NaCl solutions containing 2 mM CPC **(B).** After topical lingual application of 20 mM 8-CPT-cAMP for 10 min the CT responses were again monitored under open circuit conditions while the tongue was first rinsed with a rinse solution R (10 mM KCl) and then with 0.1, 0.3 and 0.5 M NaCl **(C)** and in NaCl solutions containing 2 mM CPC **(D)** or 2 mM CPC + 5 μM Bz **(E)**. **(F)** Shows the mean normalized tonic CT responses for various concentrations of NaCl, NaCl + CPC and ((NaCl)-(NaCl + CPC)). Each bar shows the mean CT response from the number of animals (*N*). For Control: 0.1–0.5 M NaCl (*N* = 12–18); Control NaCl + CPC (*N* = 10–13); post 8-CPT-cAMP 0.1–0.5 M NaCl (*N* = 9–12); NaCl + CPC (*N* = 5–9). No changes in the Bz-insensitive NaCl CT response ((NaCl)-(NaCl + CPC)) were observed (P>0.05) at any NaCl concentration (Fig 2F; grey bars). *p = 0.0001 with respect to control. **(G)** Shows the comparative changes in Bz-sensitive NaCl CT responses in 19–23 day old and adult (60+ day old) rats. The values of mean normalized ENaC-dependent NaCl tonic CT response magnitudes before and after 20 mM 8-CPT-cAMP treatment in 19–23 day old rats were taken from Fig F (filled bars). The relative values of ENaC-dependent NaCl tonic CT response magnitudes in control adult rats are shown in hatches bars (*N* = 3). *P = 0.0001 with respect to control adult rats.

Under open-circuit conditions, in 19–23 day old rats, the CT response increased with increasing NaCl concentration from 0.1 to 0.5 M (Fig 2A and 2F). Adding CPC to each NaCl stimulus eliminated most of NaCl CT response (Fig 2B and 2F). This suggests that in 19–23 day old rats a small number of functional apical ENaCs are present in FF TRCs that contribute to a small NaCl CT response just above the rinse baseline level. Following topical lingual application of 20 mM 8-CPT-cAMP for 10 min, the response to NaCl was significantly enhanced relative to control (Fig 2C and 2F). The enhanced response was partially blocked by CPC (Fig 2D and 2F), but was inhibited to the rinse baseline level in the presence of CPC + Bz (Fig 2E). This suggests that in 19–23 day old rats, the cAMP-induced enhanced NaCl CT response is the result of ENaC activation. The post-8-CPT-cAMP NaCl CT response (Fig 2C), consisted of two components, an ENaC-dependent (Bz-sensitive) component and a Bz-insensitive but CPC-sensitive component. No change in the Bz-insensitive NaCl CT response ((NaCl)-(NaCl + CPC)) was observed at any NaCl concentration (Fig 2F; grey bars).

In 19–23 day old rats treated with 20 mM 8-CPT-cAMP, the mean normalized ENaC-dependent tonic CT response at 0.1 M NaCl was not different from the ENaC-dependent tonic CT response at 0.1 M NaCl in control adult rats (Fig 2G). However at 0.3 and 0.5 M NaCl, post-8-CPT-cAMP-dependent NaCl tonic CT responses in 19–23 day old rats remained significantly lower than ENaC-dependent NaCl tonic CT responses in control adult rats (Fig 2G).

In contrast to 8-CPT-cAMP, topical lingual application of 20 mM 8-CPT-cGMP for 10–15 min had no effect on the NaCl CT responses in 19–23 day old rats (data not shown). These results indicate that NaCl CT responses are specifically enhanced by cAMP.

**Fig 3:**
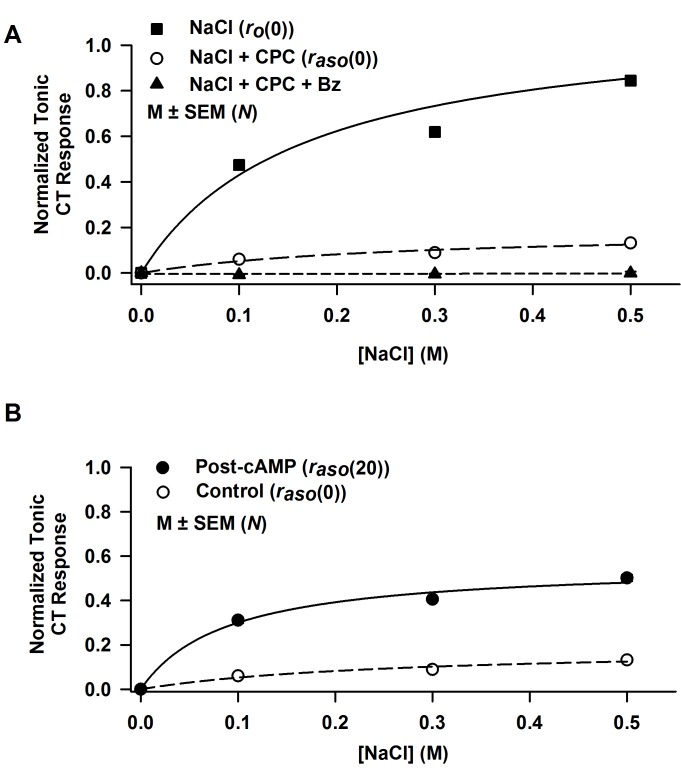
Effect of 8-CPT-cAMP on ENaC-dependent NaCl CT responses in 19–23 day old rats. **(A)** Mean normalized tonic CT response under open-circuit conditions (filled squares) to NaCl (*r*_*o*_(0)) at zero 8-CPT-cAMP (*N* = 12–18). Since *r*_*o*_(0) = *r*_*aso*_(0) + *r*_*aio*_(0), the solid line through the data points is the sum of the regression curves for *r*_*aso*_(0) and *r*_*aio*_(0). Mean normalized tonic CT response under open-circuit conditions (open circles) to NaCl + CPC (*r*_*aso*_(0)) at zero 8-CPT-cAMP (*N* = 10–13). Curve (long dashes) is the regression curve. CT response under open-circuit conditions (closed triangles) to NaCl + CPC + Bz at zero 8-CPT-cAMP (*N* = 3). Curve (short dashes) is the linear regression line. **(B)** Mean ENaC-dependent part of the CT response as a function of NaCl concentration under open circuit conditions in 19–23 day old rats before (r_aso_(0); open circles) and following exposure to 20 mM CPT-cAMP (r_aso_(20); filled circles). The curves are the least squares fits to the data using Eq 1 with *φ* = 0. The dotted line represents the pre-cAMP control curve and the solid curve represents the post 20 mM 8-CPT-cAMP curve. Each NaCl solution contained 2 mM CPC to block the Bz-insensitive part of the response. For r_aso_(0), *N* = 10 (0.1 M NaCl), *N* = 13 (0.3 M NaCl) and *N* = 10 (0.5 M NaCl). For r_aso_(20), *N* = 5 (0.1 M NaCl), *N* = 9 (0.3 M NaCl) and *N* = 10 (0.5 M NaCl).

Under control conditions, in 19–23 day old rats, the Bz-sensitive component (NaCl + CPC; *r*_*aso*_(0)) was a minor component at all NaCl concentrations tested (Fig 3A; ○). In the presence of both Bz and CPC the CT response decreased to the baseline rinse level (Fig 3A; ▲). This shows that the solid curve for the whole NaCl CT response (*r*_*o*_(0); Eq 3) in Fig 3A (■) is, therefore, the sum of curves derived for *r*_*aso*_(0) using Eq 1 representing the Bz-sensitive component, and *r*_*aio*_(0) using Eq 2 representing the amiloride- and Bz-insensitive component.

The changes in the mean open-circuit CT response to NaCl + CPC (*r*_*aso*_) as a function of NaCl concentration before and after the topical lingual application of 20 mM 8-CPT-cAMP in 19–23 day old rats are shown in Fig 3B. The data were fitted to Eq 1. The values of the maximal ENaC-dependent response (*r*_*asmc*_) and *K*_*asc*_ for the control curve and for the post-8-CPT-cAMP curve are shown in Table 2. Following 8-CPT-cAMP treatment *r*_*asm*_ increased (p = 0.0001) and *K*_*as*_ decreased.

**Table 2:** Effect of 8-CPT-cAMP on the parameters *r*_*as*_, *K*_*as*_, *r*_*ai*_ and *K*_*ai*_ of the NaCl CT responses in 19–23 day old rats.

**8-CPT-cAMP (mM)**	***r*_*as*_**	***K*_*as*_ (M)**	***R*^*2*^**
0	0.191 ± 0.043	0.263 ± 0.08	0.97
20	0.565 ± 0.050*	0.088 ± 0.030	0.98
	***r***_***ai***_	***K***_***ai***_ **(M)**	
0 or 20	0.952 ± 0.073	0.152 ± 0.034	0.98

Values are mean ± SEM (*N*) from Fig 2

* P = 0.0001 (unpaired)

As shown in Fig 2F (grey bars), relative to control, 20 mM 8-CPT-cAMP treatment did not produce any significant changes in the Bz-insensitive NaCl CT response ((NaCl)-(NaCl + CPC)). Therefore, the normalized values of the Bz-insensitive NaCl CT response at each NaCl concentration before and after 8-CPT-cAMP treatment were combined to estimate the parameters in Eq 2 for *r*_*aio*_. Using the least squares fit of the combined data points gave *r*_*aim*_ and *K*_*ai*_ values also shown in Table 2. These results indicate that the cAMP effects on the NaCl CT response in 19–23 day old rats are independent of the Bz-insensitive component of the CT response.

### Concentration-dependence of 8-CPT-cAMP on *r*_*asm*_ and *K*_*as*_

**Fig 4:**
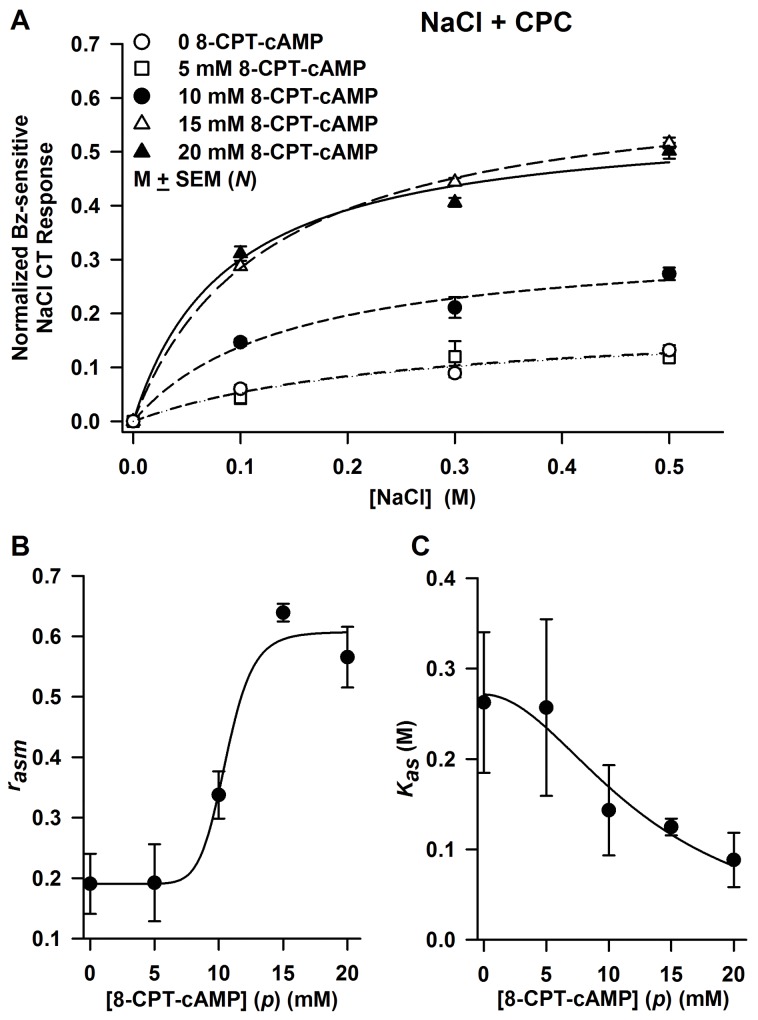
Concentration-dependence of 8-CPT-cAMP on *r*_*asm*_ and *K*_*as*_. **(A)** Shows open-circuit Bz-sensitive NaCl CT response curves in 19-23d old rats after exposing their tongues to 0 (*N* = 10–13), 5 mM (*N* = 3), 10 mM (*N* = 3), 15 mM (*N* = 3), and 20 mM (*N* = 5–9) 8-CPT-cAMP. Each data point represents the mean ± SEM of *N*, the number of animals in each group. The non-linear regression curves were obtained by fitting to Eq 1. The goodness of fit parameter R^2^ for each of the five curves varied between 0.96 and 0.99. **(B)** Shows the 8-CPT-cAMP concentration (*p*) dependence of the ENaC-dependent maximum CT response (*r*_*asm*_) found from the regression curves in Fig 4A. The solid curve is the least squares fit to Eq 8 with *r*_*asmo*_ *=* 0.190 ± 0.043_,_ a = 0.417 ± 0.081, k = 10.6 ± 1.5 mM, *n* = 10.0 ± 3.1, and R^2^ = 0.98. **(C)** Shows 8-CPT-cAMP concentration dependence of the constant *K*_*as*_ (in moles/liter). *K*_*as*_ values were obtained from regression analysis of the five curves in Fig 4A each corresponding to a different 8-CPT-cAMP concentration. The curve is the regression line (R^2^ = 0.95) with parameters selected to yield the best fit to Eq 9, i.e. *b* = 0.271 ± 0.025 M, *q* = 12.954 ± 2.143 mM, and *n* = 1.946 ± 0.686.

In 19–23 day old rats, CT responses to NaCl + CPC were obtained before and after topical lingual application of 5, 10, 15, and 20 mM 8-CPT-cAMP (Fig 4A). The data were fitted to Eq 1 and yielded values of *r*_*asm*_ and *K*_*as*_ corresponding to a particular 8-CPT-cAMP concentration, *p*. The Bz-sensitive maximum CT response, *r*_*asm*_, was a saturating sigmoidal function of 8-CPT-cAMP concentration as *p* → ∞ (Fig 4B). The maximum increase in *r*_*asm*_ relative to *r*_*asmc*_, was found by least squares fit of the data to Eq 8, where it is expressed by parameter *a*, whose value was 0.417 ± 0.081 (Table 3). At zero 8-CPT-cAMP *r*_*asm*_ was 0.191 ± 0.043 (from Fig 3B). The ratio *a/r*_*asmc*_ is 2.18 (218%) and represents the 8-CPT-cAMP-induced increase in *r*_*asm*_ relative to *r*_*asmc*_. This ratio is slightly less than the value 2.96 (0.565/0.191) calculated from values shown in Table 2. In the ENaC channel model [51], *r*_*asm*_ is proportional to the density of functional apical ENaCs. This suggests that in 19–23 day old rats, 8-CPT-cAMP increases the functional ENaC density by 218%. The parameter *k* expresses the 8-CPT-cAMP concentration at which the increase in *r*_*asm*_ relative to *r*_*asmc*_ is one-half maximal (Table 3). The large *n* value (Table 3) is an expression of the high threshold 8-CPT-cAMP concentration applied to the tongue (about 10 mM) before a cAMP effect is observed. As cAMP concentration (*p*) increases *K*_*as*_ decreases (Fig 4C). The curve is the least squares fit to Eq 9.

**Table 3:** Effect of 8-CPT-cAMP on the parameters *r*_*asm*_ and *K*_*asc*_ of the NaCl CT responses in 19–23 day old rats.

Fig 4B^†^	*a*	*a/r*_*asmc*_	*K*_*asc*_ (mM)	*n*	*R*^*2*^
NaCl + CPC	0.417 ± 0.081	2.18	10.6 ± 1.5	10.0 ± 3.1	0.98

^†^ The ENaC-dependent maximum NaCl CT response (*r*_*asm*_) relative to *r*_*asmc*_ was found by least squares fit of the data in Fig 4B to Eq 8, where it is expressed by a parameter *a*

### 8-CPT-cAMP increases the voltage sensitivity of the Bz-sensitive NaCl CT response

**Fig 5:**
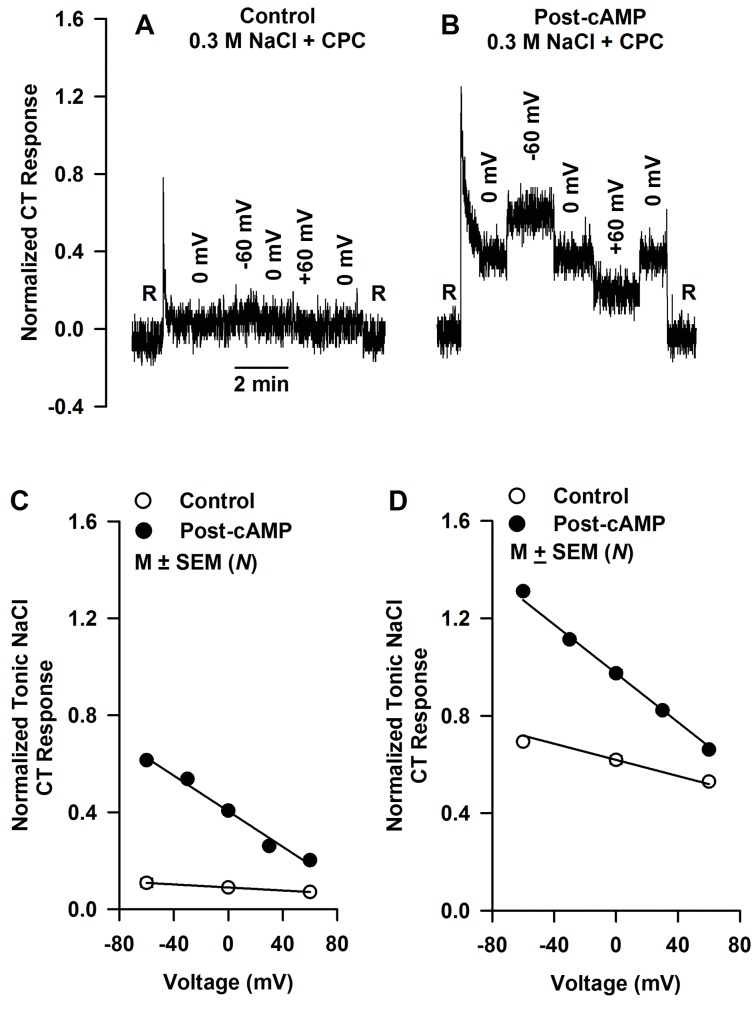
Effect of applied translingual voltage on the NaCl CT response. Shows a representative CT response in a 19 day old rat to 0.3 M NaCl + 2 mM CPC at -60, 0 and 60 mV before **(A)** and after topical lingual application of 20 mM 8-CPT-cAMP for 10 min **(B)**. **(C)** Shows the mean variation in the Bz-sensitive NaCl CT response (*r*_*as*_) to 0.3 M NaCl + 2 mM CPC with applied voltage in 19–23 day old rats under control conditions (open circles; *N* = 3–13) and post-20 mM 8-CPT-cAMP (closed circles; *N* = 3–9). *N* represents the number rats in each group.

In a 19 day old rat, the ENaC-dependent open-circuit CT response to 0.3 M NaCl was small relative to baseline and virtually insensitive to the applied voltages (Fig 5A). Following treatment with 20 mM 8-CPT-cAMP for 10 min, the same rat demonstrated a significantly increased NaCl CT response and marked sensitivity to voltage (Fig 5B). The ENaC-dependent NaCl CT response varied linearly with applied voltage between -60 mV and 60 mV (Fig 5C) [21, 55]. Treatment of the tongue with 8-CPT-cAMP increased both *r*_*aso*_ (the open-circuit response to NaCl + CPC), but also the rate of increase in *r*_*as*_ per unit change in translingual epithelial voltage (response conductance, *κ*_*as*_). For each condition *r*_*as*_ is described by Eq 5, and the calculated parameters are given in Table 4. In 19–23 day old rats, 20 mM 8-CPT-cAMP treatment increased *κ*_*as*_ by a factor of 11.7 (0.00367/0.000313; Table 3).

**Table 4:** Effect of 8-CPT-cAMP on the response conductance (*κ*_*as*_) of Bz-sensitive NaCl CT responses in 19–23 day old rats.

NaCl	8-CPT-cAMP	*κ*_*as*_ (mV)^-1^	*r*_*as*_	δ
0.3 M	0	3.13 x 10^−4^ ± 0.3 x 10^−4^	0.0894 ± 0.001	0.14
	20	^†^36.7 x 10^−4^ ± 2.0 x 10^−4^	*0.404 ± 0.009	0.93

The values are mean ± SEM (3)

* P = 0.0001

^†^P = 0.0002 (unpaired)

Since *r*_*asm*_, and *K*_*as*_ are known from open-circuit analysis (Fig 4B and 4C), the response conductance (Eq 6) yields values of δ (0.14) under control conditions and (0.93) post-20 mM 8-CPT-cAMP (Table 4). Thus, only 14% of the clamp voltage is dropped across functional apical membrane ENaCs under control conditions and 93% of the voltage is dropped across the significantly increased number of functional ENaCs post-20 mM 8-CPT-cAMP treatment.

### Effect of 8-CPT-cAMP and 8-CPT-cGMP on behavioral responses to NaCl in the developing rats

**Fig 6:**
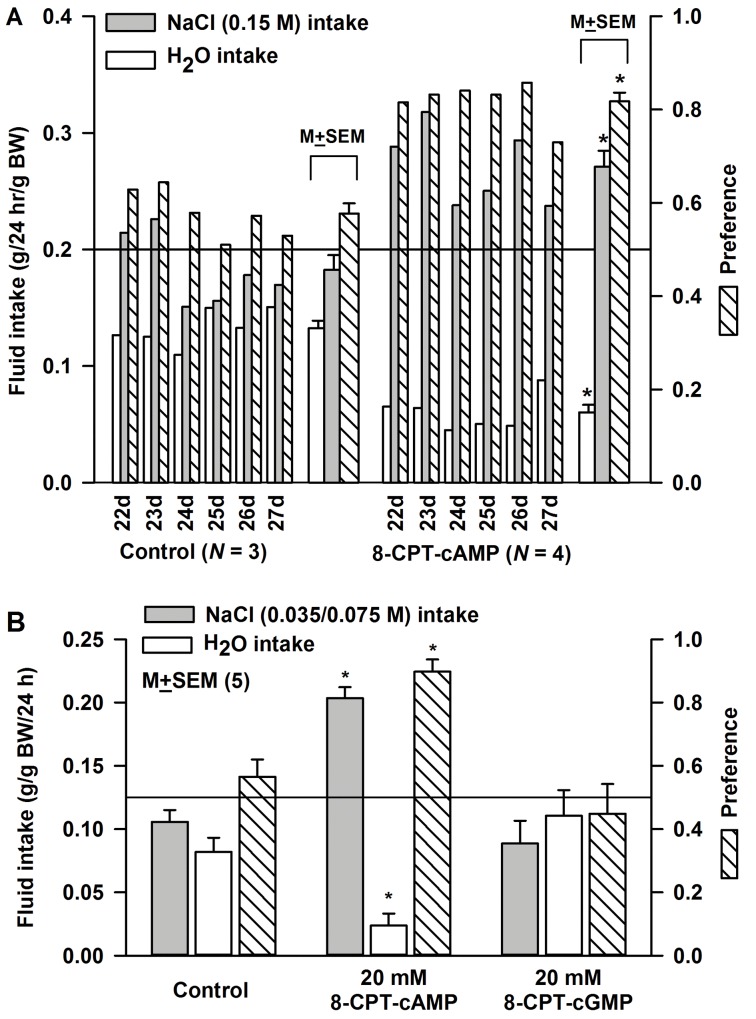
Fluid intake and NaCl preference in developing rats. **(A)** Rat (21 day old) tongues were treated with either H_2_O (Control; *N* = 3) or 20 mM 8-CPT-cAMP (Post-8-CPT-cAMP; *N* = 4) for 20 min. The 3 control and 4 treated rats were then transferred to separate cages and were given choice between two bottles, one containing H_2_O and the other containing 0.15 M NaCl. Their fluid intake was measured every day between postnatal day 22 and 27 and expressed as g/g body weight (BW)/24h. The bars under the brackets represent the mean ± SEM values of fluid intake and NaCl preference over the 6 day period in control (*N* = 3) and 8-CPT-cAMP treated rats (*N* = 4). Horizontal line denotes NaCl preference ratio of 0.5. In 8-CPT-cAMP treated rats, there was a decrease in H_2_O intake (*p = 0.0006) and an increase in NaCl intake (*p = 0.006) relative to control rats. The mean preference in control and 8-CPT-cAMP treated rats was 0.577 ± 0.02 and 0.818 ± 0.018, respectively (*p = 0.0004). **(B)** Rat (21d old) tongues were treated with either H_2_O (Control; *N* = 5) or 20 mM 8-CPT-cAMP (Post-8-CPT-cAMP; *N* = 5) or 20 mM 8-CPT-cGMP (Post-8-CPT-cGMP; *N* = 5) for 20 min. The control and treated rats were then transferred to separate cages and were given choice between two bottles, one containing H_2_O and the other containing 0.035 or 0.075 M NaCl. Their fluid intake and NaCl preference was measured every day between postnatal day 22 and 27. Only rats treated with 8-CPT-cAMP demonstrated a significant increase in NaCl preference. *p = 0.0001 (*N* = 5).

Two bottle/24h NaCl preference tests were performed on 21 day old rats following topical lingual treatment with H_2_O or 20 mM 8-CPT-cAMP for 20 min (Fig 6A). The control and treated rats were housed in two separated cages and were given access to H_2_O and 0.15 M NaCl and their combined fluid intake was measured each day every 24h. In control rats, the mean NaCl preference for a period of 6 days (postnatal day 22–27) was 0.58 ± 0.02 (*N* = 3). In 8-CPT-cAMP treated rats (*N* = 4), there was a significant decrease in H_2_O intake and a significant increase in NaCl intake, and the mean NaCl preference for the 6 day period was 0.82 ± 0.018 (P = 0.0001; *N* = 4).

In a separate experiment, rat (21 day old) tongues were treated with H_2_O (*N* = 5), 20 mM 8-CPT-cAMP (*N* = 5) or 20 mM 8-CPT-cGMP (*N* = 5) for 20 min. The above 3 groups of rats were housed in three separated cages and were given a choice between H_2_O and 0.035 M NaCl or H_2_O and 0.075 M NaCl. Their combined fluid intake was measured each day. For the group of rats whose tongues were treated with H_2_O, 8-CPT-cAMP or 8-CPT-cGMP the mean combined NaCl preference at 0.035 and 0.075 M NaCl was 0.56 ± 0.055, 0.90 ± 0.039 (P = 0.0001), and 0.45 ± 0.094, respectively.

### Effect of 8-CPT-cAMP and 8-CPT-cGMP on behavioral responses to NaCl in adult rats

**Fig 7:**
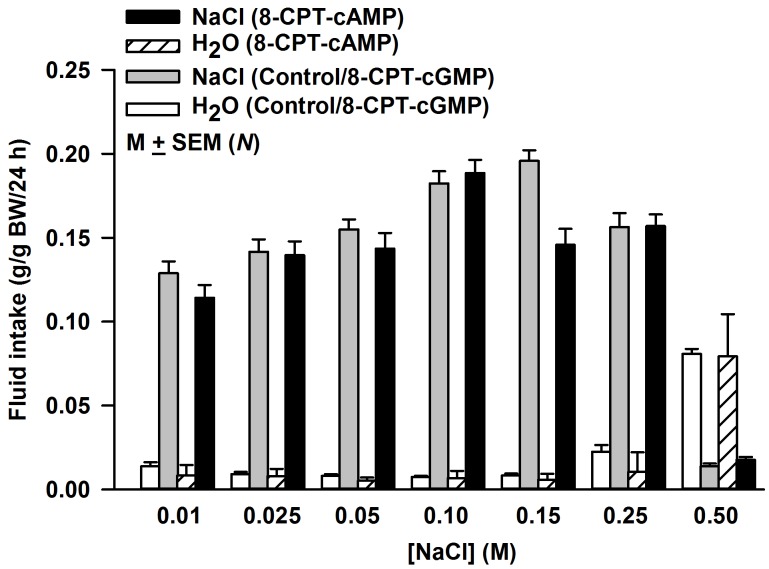
Effect of lingual application of 8-CPT-cAMP or 8-CPT-cGMP on fluid intake in adult rats. Adult (60+ day old) rat tongues were treated with either H_2_O (Control; *N* = 15) or 20 mM 8-CPT-cAMP (Post-8-CPT-cAMP; *N* = 15) or 20 mM 8-CPT-cGMP (*N* = 14). The rats were given a choice between two bottles, one containing H_2_O and the other containing varying NaCl concentrations (0.01–0.5 M NaCl) and their fluid intake was measured every day. No significant difference in the intake of H_2_O or NaCl was observed between control and 8-CPT-cGMP treated rats at different NaCl concentrations. Therefore, the intake of H_2_O and NaCl was presented as the mean intake observed in control and 8-CPT-cGMP treated rats (*N* = 30). The mean intake of NaCl at 0.15 M NaCl (grey bars) was significantly different from the intake at 0.01, 0.025, 0.05, 0.10, 0.25 and 0.5 M NaCl with P values of 0.0001, 0.0001, 0.0001, 0.1657, 0.0004, and 0.0001, respectively (*N* = 30; unpaired). In 8-CPT-cAMP treated rats the intake at 0.1 M NaCl (black bars) was significantly different from the intake at 0.01, 0.025, 0.05, 0.15, 0.25 and 0.5 M NaCl with P values of 0.0001, 0.0002, 0.0010, 0.0020, 0.0058, and 0.0001, respectively (*N* = 14; unpaired).

Two bottle/24h NaCl preference tests were performed in adult rats following topical lingual treatment with H_2_O or 20 mM 8-CPT-cAMP or 20 mM 8-CPT-cGMP for 20 min (Fig 7). The control and treated rats were housed individually in separated cages and were given access to H_2_O and varying concentrations of NaCl (0.01–0.5 M) and their fluid intake was measured each day. There was no difference between H_2_O intake and NaCl intake between 8-CPT-cGMP treated rats and control rats. Accordingly, the data from both groups was combined. In control rats and in rats treated with 20 mM 8-CPT-cGMP, the NaCl intake increased linearly between 0.01 and 0.15 M NaCl (Fig 7; grey bars). At 0.25 and 0.5 M NaCl the NaCl intake was significantly reduced relative to 0.15 M NaCl. In rats treated with 20 mM 8-CPT-cAMP, the NaCl intake increased linearly between 0.01 and 0.10 M NaCl (Fig 7; black bars). At 0.15, 0.25 and 0.5 M NaCl the NaCl intake was significantly reduced relative to 0.1 M NaCl. These results suggest that in both young and adult rats, the behavioral responses to NaCl are specifically modulated by 8-CPT-cAMP and not by 8-CPT-cGMP.

### Age-dependent increase in ENaC expression

**Fig 8:**
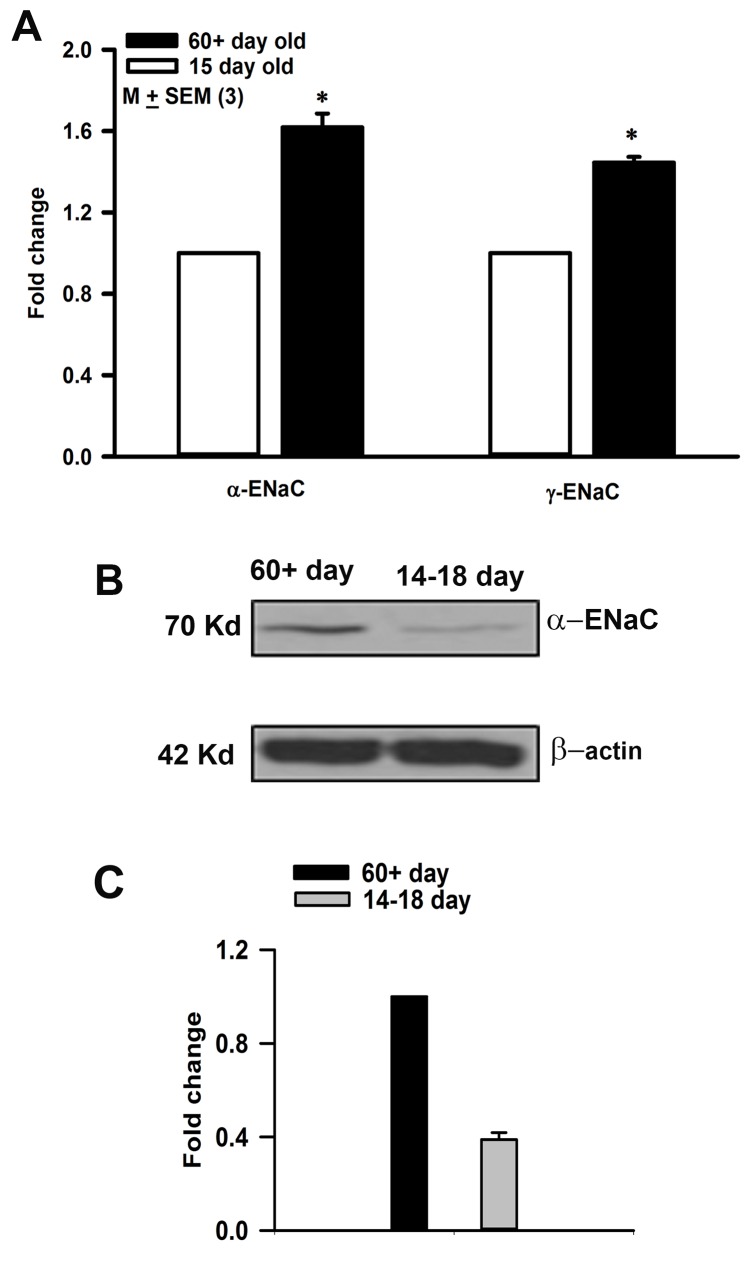
Age-dependent increase in ENaC expression. **(A)** CV papillae from naïve rats at different age were collected for Q-PCR to determine the age dependent mRNA level of α-ENaC and γ-ENaC. The results showed the mRNA levels of both subunits is higher in adult rat CV papillae compared with the pups. **(B)** For Western blot studies, CV papillae were pooled from fifteen 14–18 day old rats and eight adult (60+ day old) rats. Forty μg total protein was used for the assay. Beta-actin was used as a protein loading control. **(C)** Shows the intensity of the α-ENaC band in 14–18 day old rats relative to adult rats from 3 different batches of CV tissues normalized to β-actin (p = 0.0001).

In the anterior taste receptive field ENaC is expressed in a subset of FF TRCs and in squamous epithelial cells. Fungiform taste buds are difficult to separate without contamination from the surrounding non-taste tissue. Accordingly, we investigated ENaC expression in pure taste buds isolated from CV taste papillae from young and adult rats. In our Q-PCR studies, α- and γ-ENaC mRNA levels were significantly higher in adult CV taste buds than in 15 day old rats (Fig 8A). Relative to adult rats, in CV taste bud lysates a significantly lower expression of α-ENaC protein was observed in 14–18 day old rats (Fig 8B). These results indicate that during development there is an age-dependent increase in ENaC expression in CV TRCs (Fig 8C). We hypothesize that a similar age-dependent increase in ENaC expression also occurs in FF TRCs.

### Spaciotemporal relationship between cAMP formation and ENaC trafficking

**Fig 9:**
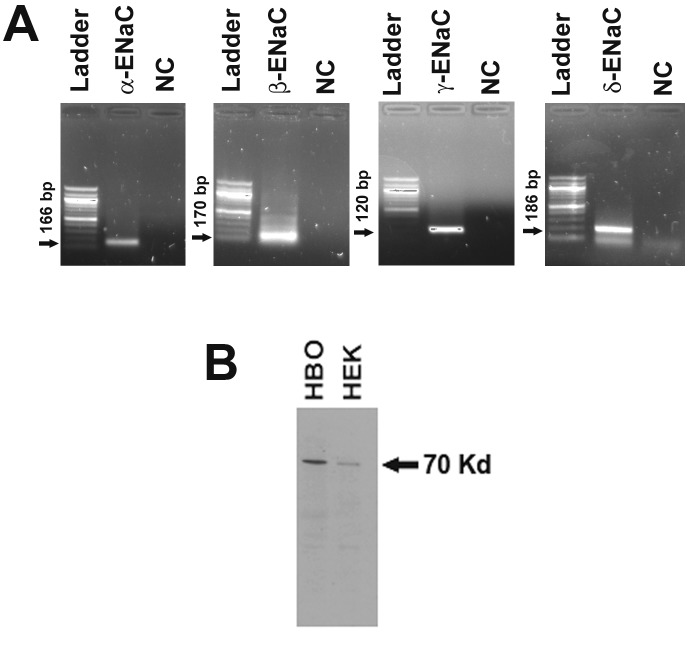
HBO cells express ENaC. **(A)** RT-PCR studies demonstrated the expression of α-, β-, γ-, and δ-ENaC subunit mRNAs in HBO cells. The arrows show the band size. **(B)** Western blot studies demonstrated the expression of δ-ENaC protein in HBO cells. HEK-293 cells were used as a positive control. The arrow show the band size.

The spaciotemporal relationship between cAMP formation and ENaC trafficking was investigated in HBO cells. In HBO cells, mRNAs for α-, β-, γ-, and δ-ENaC subunits were detected by RT-PCR (Fig 9A) and δ-ENaC protein by Western blot (Fig 9B).

**Fig 10:**
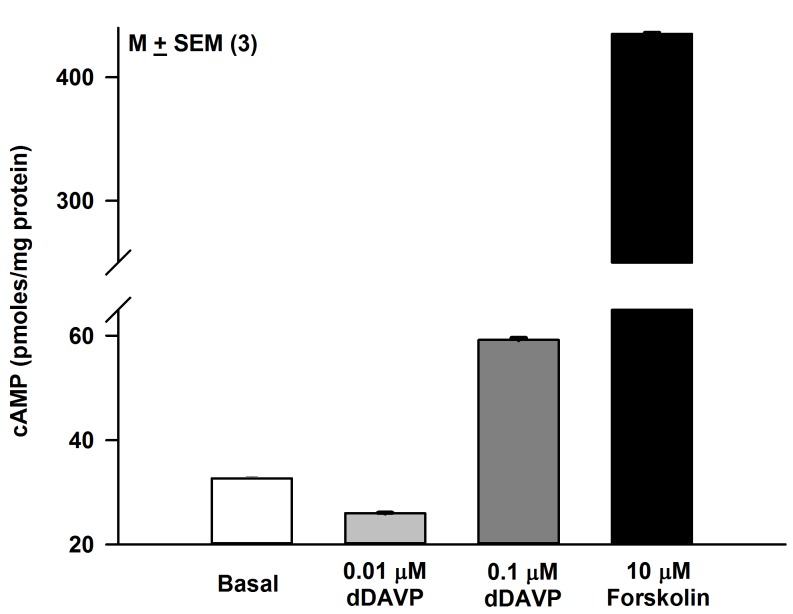
Effect of dDAVP on intracellular cAMP formation in HBO cells. Treating HBO cells for 10 min with dDAVP (0.1 μM) increased intracellular cAMP. Forskolin (10 μM), a known activator of adenylyl cyclase was used as a control. The values represent mean ± SEM of triplicate measurements.

**Fig 11:**
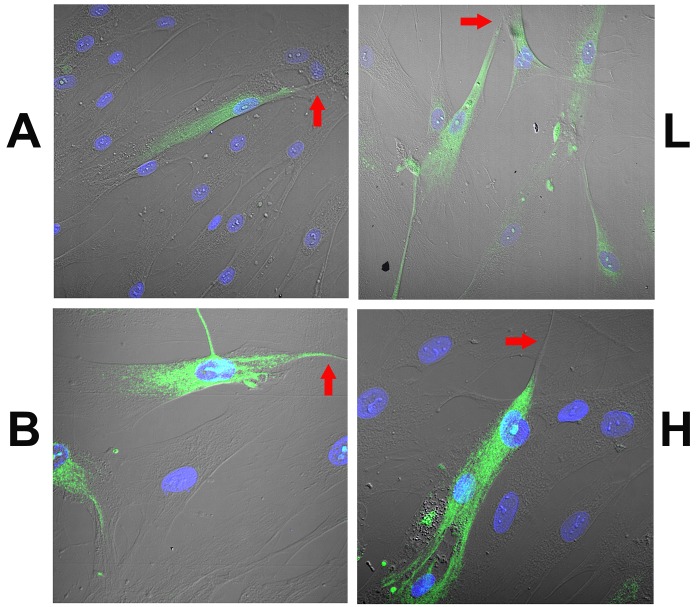
Localization of δ-ENaC in control HBO cells. The figure shows the overlay of the transmitted (DIC) image, DAPI labelled cell nuclei (blue) and δ-ENaC binding to HBO cells (green). **(Panel A)** Shows low resolution images **(L)** of δ-ENaC antibody binding to HBO cells under control conditions. The δ-ENaC-antibody binding was observed only in a subset of HBO cells. In 14 images, out of 113 HBO cells examined, 27 cells (23.9%) were positive for δ-ENaC. **(Panel B)** Shows high resolution images **(H)** of δ-ENaC antibody binding to a subset of control HBO cells. The δ-ENaC antibody binding was observed mainly in the cytosolic compartment with much less binding in the apical compartment of HBO cells (arrows).

**Fig 12:**
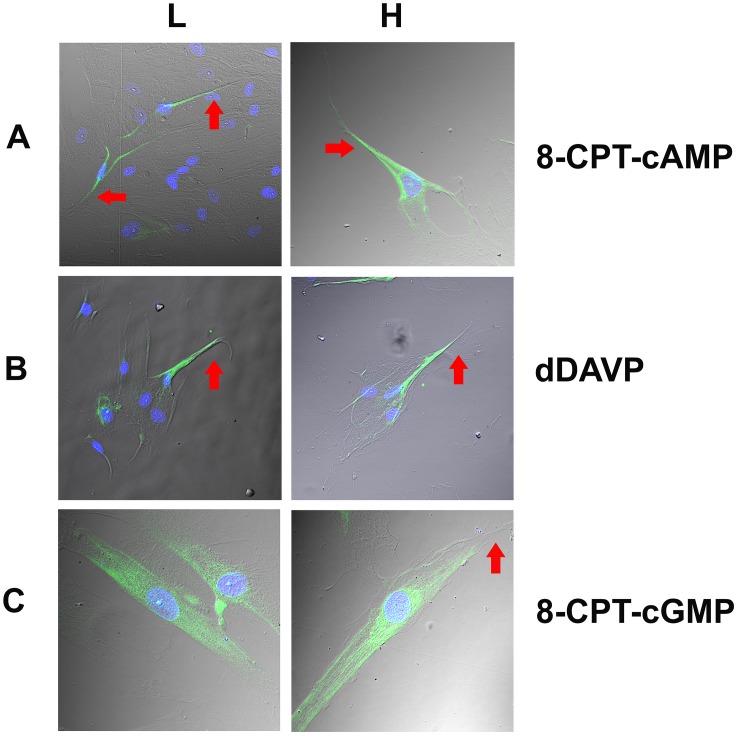
Spaciotemporal relationship between 8-CPT-cAMP, dDAVP and 8-CPT-cGMP treatment and δ-ENaC trafficking in HBO cells. The figure shows the overlay of the transmitted (DIC) image, DAPI labelled cell nuclei (blue) and δ-ENaC binding to HBO cells (green). **(Panel A)** Shows δ-ENaC antibody binding to a subset of HBO cells treated with 10 μM 8-CPT-cAMP for 10 min in a representative low resolution **(L)** and a high resolution **(H)** image. The δ-ENaC antibody binding was observed mainly in the apical compartment with much less binding in the cytosolic compartment of HBO cells (arrows). **(Panel B)** Shows δ-ENaC antibody binding to a subset of HBO cells treated with 10 nM dDAVP for 10 min in a representative low resolution **(L)** and a high resolution **(H)** image. Similar to HBO cells treated with 8-CPT-cAMP (Panel A), the δ-ENaC antibody binding was observed mainly in the apical compartment with much less binding in the cytosolic compartment in a subset of HBO cells (arrows). **(Panel C)** Shows δ-ENaC antibody binding to a subset of HBO cells treated with 10 μM 8-CPT-cGMP for 10 min in a representative low resolution **(L)** and a high resolution **(H)** image. Similar to control HBO cells (Fig 11), δ-ENaC antibody binding was observed mainly in the cytosolic compartment with much less binding in the apical compartment of HBO cells (arrows).

HBO cells treated with dDAVP for 10 min produced a dose-dependent increase in intracellular cAMP (Fig 10). In control HBO cells δ-ENaC antibody binding was observed in a subset of cells (Fig 11; Panel A). The antibody binding was observed mainly in the cytosolic compartment (Fig 11; Panel B). Out of 113 cells examined, 27 cells were δ-ENaC positive (23.9%). In contrast, incubating HBO cells with 10 μM 8-CPT-cAMP (Fig 12; Panel A) or 10 nM dDAVP (Fig 12; Panel B) for 10 min, resulted in trafficking of δ-ENaC from the cytosolic compartment to the apical compartment of a subset of HBO cells. However, similar to the control HBO cells (Fig 11), in HBO cells treated with 10 μM 8-CPT-cGMP for 10 min, the δ-ENaC antibody binding was observed mainly in the cytosolic compartment of a subset of HBO cells (Fig 12; Panel C).

**Fig 13:**
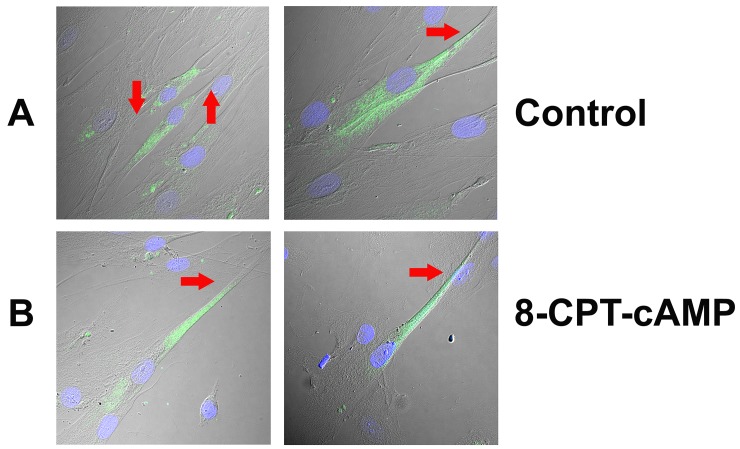
Spaciotemporal relationship between cAMP and γ-ENaC trafficking in HBO cells. The figure shows the overlay of the transmitted (DIC) image, DAPI labelled cell nuclei (blue) and γ-ENaC binding to HBO cells (green). (**Panel A**) Shows γ-ENaC antibody binding to a subset of control HBO cells. The γ-ENaC binding was observed mainly in the cytosolic compartment with much less binding in the apical compartment of HBO cells. (**Panel B**) Shows γ-ENaC antibody binding to a subset of HBO cells treated with 10 μM 8-CPT-cAMP for 10 min. The γ-ENaC binding was observed mainly in the apical compartment with much less binding in the cytosolic compartment of HBO cells.

We also observed γ-ENaC antibody binding to a subset of HBO cells, mainly in the cytosolic compartment (Fig 13; Panel A). Treating HBO cells with 10 μM 8-CPT-cAMP for 10 min induce trafficking of γ-ENaC subunit from the cytosolic compartment to the apical compartment (Fig 13; Panel B). These results show that dDAVP and cAMP induce trafficking of δ and γ ENaC subunits from the cytosolic compartment to the apical membranes of salt sensing HBO cells. We hypothesize that dDAVP and cAMP also induce trafficking of α and β ENaC subunits to form functional ENaCs in the apical membrane of HBO cells.

In addition to γ and δ ENaC subunit trafficking, dDAVP and 8-CPT-cAMP also induced changes in the cell shape, resulting in a slight narrowing of HBO cells.

### Effect of AVP and dDAVP on NaCl CT responses in adult rats

**Fig 14:**
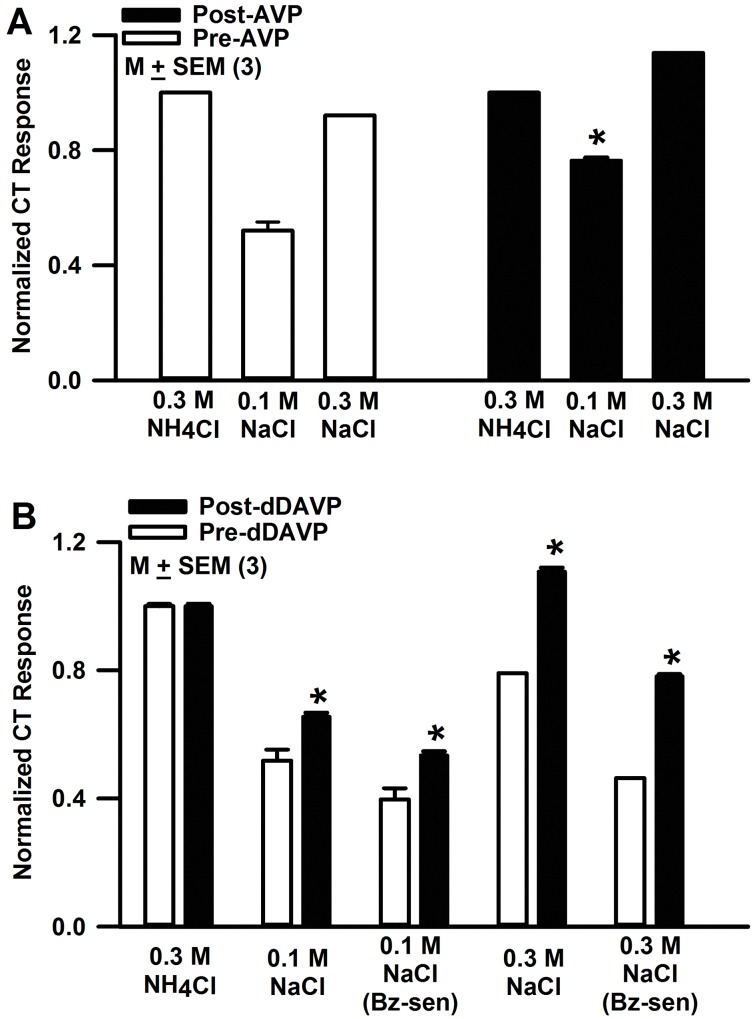
Effect of AVP and dDAVP on NaCl CT responses in adult rats. **(A)** In adult rats CT responses to 0.1 and 0.3 M NaCl were measured before and 20 min after tail vein injection of 1 nano moles AVP/Kg BW. The figure shows the mean normalized tonic NaCl CT responses in control and AVP-treated rats. *P = 0.0015 with respect to control. **(B)** In another set of rats, CT responses to 0.1 and 0.3 M NaCl were measured in the absence and presence of 5 μM Bz, before and 20 min after tail vein injection of 0.1 nano moles dDAVP/Kg BW. The values are mean normalized tonic NaCl CT responses from 3 rats in each group. *P = 0.02 with respect to control. The CT responses were normalized to 0.3 M NH_4_Cl.

In adult rats, a single tail vein injection of AVP (1 nano moles/Kg BW) enhanced the CT response to 0.1 and 0.3 M NaCl 20 min post-AVP injection (Fig 14A). In additional rats, a single tail vein injection of dDAVP (0.1 nano moles/Kg BW) enhanced the CT response to 0.1 and 0.3 M NaCl 20 min post-dDAVP injection. The increase in NaCl CT response was specifically due to an increase in the ENaC-dependent component of the NaCl CT response (Fig 14B).

### Effect of dDAVP on ENaC expression and trafficking in rat FF TRCs

**Fig 15:**
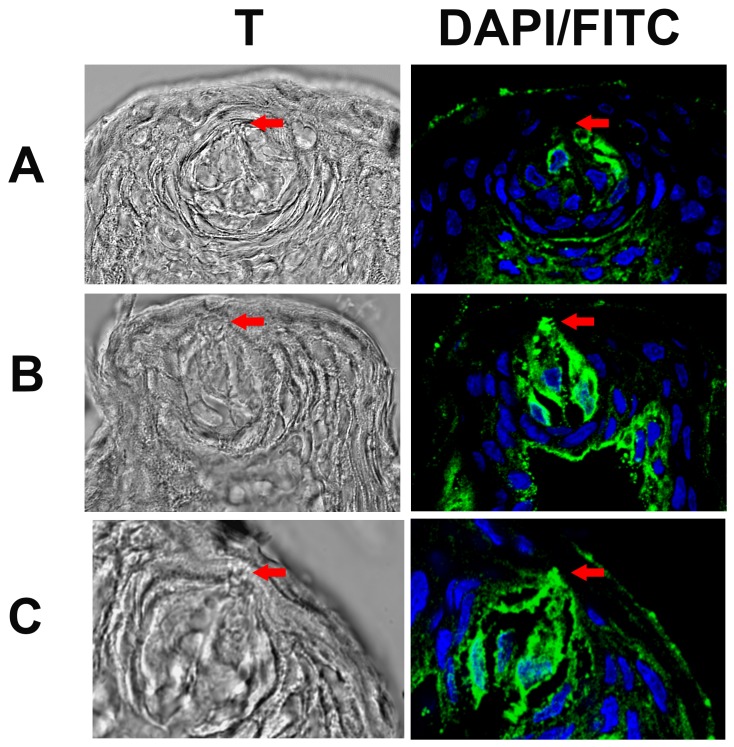
Effect of dDAVP on γ-ENaC expression and trafficking in FF TRCs. The confocal images show the transmitted images (T) and the overlay of DAPI labelled cell nuclei (blue) and γ-rENaC antibody binding (green). The figure shows γ-rENaC antibody binding to FF taste bud cells in saline injected 15 day old rat **(A)** and dDAVP injected 15 day old rat 24h post injection **(B and C)**. Relative to control rats, in dDAVP injected rats **(B and C),** there is more intense labelling of γ-ENaC antibody and γ-ENaC is translocated to the apical membranes of FF TRCs (arrows).

In dDAVP-injected 15 day old rats the γ-ENaC antibody binding was observed both in the cytoplasmic compartment and in the apical membranes of a subset of FF TRCs 24h post injection (Fig 15B and 15C; arrows). In rats injected with saline, the antibody binding was seen mainly in the cytosolic compartment in a subset of FF TRCs (Fig 15A). In addition, the γ-ENaC binding was more intense in dDAVP-injected rats relative to control rats. This indicates that dDAVP administration enhances γ-ENaC expression in FF TRCs and induces trafficking of γ-ENaC from cytosolic compartment to the apical membrane.

### Age-dependent increase in V2R expression

**Fig 16:**
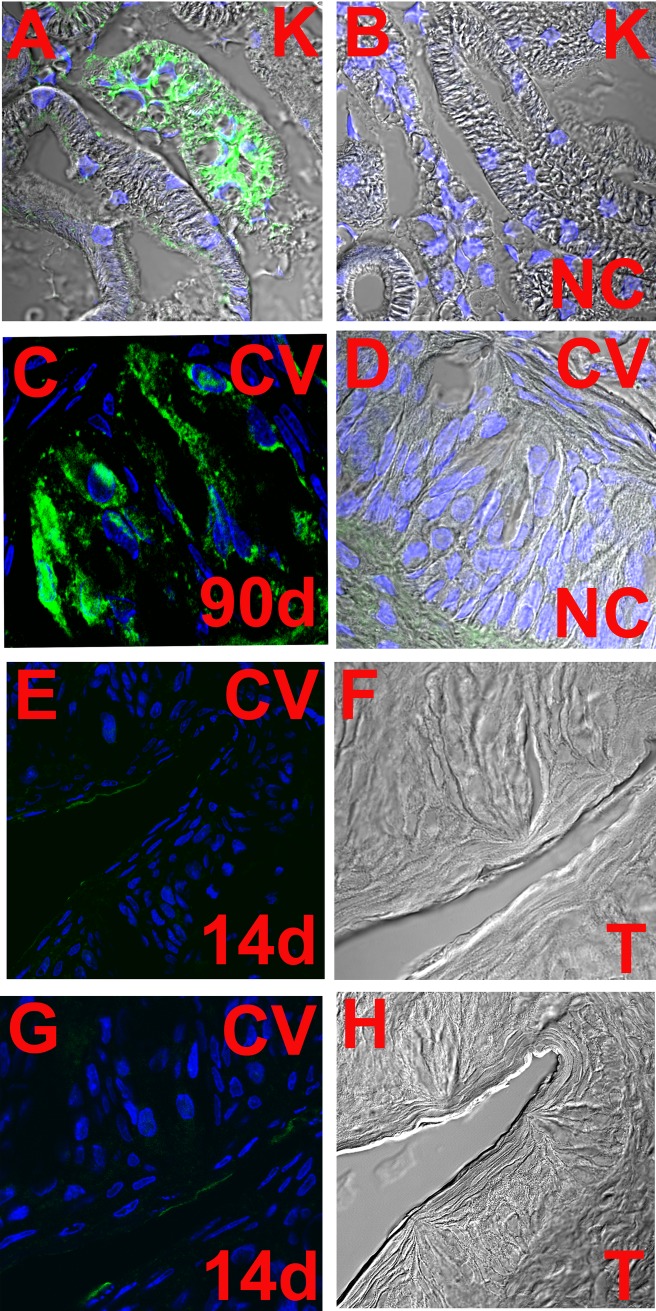
Age-dependent increase in V2R expression in CV taste bud cells. The figure shows V2R antibody binding in kidney collecting duct cells **(A)** and kidney negative control (NC) without primary antibody **(B)**. The confocal images are overlay of transmitted image, nuclei labelled with DAPI (blue) and secondary antibody IgG-CFL 488 binding (green). V2R antibody binding is shown in adult (90+ day old) rat CV taste bud cells with overlay of DAPI and green fluorescence **(C).** The corresponding NC without primary antibody is an overlay of transmitted image, DAPI and green fluorescence **(D)**. The V2R antibody binding is shown in CV taste bud cells from 14 day old rat pups (**E** and **G**). The confocal images are overlay of DAPI and green fluorescence. The corresponding transmitted images (T) are shown in (**F** and **H**).

In adult rats, V2R antibody demonstrated specific binding to kidney cortical collecting duct cells (Fig 16A) and to a subset of CV taste bud cells (Fig 16C). In CV TRCs V2R labeling was predominantly intracellular with some staining in the basolateral membrane domains (Fig 16C) [61]. While in adult rats, a subset of CV TRCs demonstrated V2R antibody binding (Fig 16C), in 14 day old rats, only a faint V2R antibody binding was observed in CV TRCs (Fig 16E and 16G). These results indicate that there is an age-dependent increase in V2R expression in TRCs.

Taken together, the above results indicate that there is a spaciotemporal relationship between AVP/dDAVP binding to V2Rs in the basolateral membrane of FF TRCs, activation of adenylyl cyclase, increase in intracellular cAMP, ENaC trafficking and the increase in the NaCl CT response and behavior.

## Discussion

In a previous study [51] tonic NaCl CT responses in rats under open circuit conditions increased in a progressive manner between 12–14 and 29–31 postnatal days. In 29–31 day old rats tonic NaCl CT responses were not different from 60+ day old rats. In addition, the sensitivity of the NaCl CT responses to applied ±60 mV across the lingual epithelium increased with age. The data were fitted to an apical Na^+^ channel kinetic model that predicted that in FF TRCs the apical Na^+^ channel density increased with age monotonically [51]. However, in this study, developmental changes in NaCl CT response and Na^+^ channel density were calculated using NaCl CT responses in the absence of amiloride or Bz. In the present study, we specifically monitored the ENaC-dependent NaCl CT response in the presence of CPC, a blocker of the amiloride-insensitive component of the NaCl CT response (Fig 1).

Our results demonstrate that in 19–23 day old rats, an incremental increase in FF TRC cAMP induced a dose-dependent increase in the magnitude of the ENaC-dependent NaCl CT response (Fig 4A). The ENaC-dependent maximum CT response (*r*_*asm*_) was a saturating sigmoidal function of 8-CPT-cAMP concentration applied to the anterior tongue (Fig 4B). The large n value (Fig 4B; Table 3) indicates that a high threshold concentration of intracellular cAMP must be reached before a cAMP effect is observed on *r*_*asm*_ (Fig 4B). With an increase in 8-CPT-cAMP concentration there was a near linear decrease in *K*_*as*_ (Fig 4C). Consistent with these results, a saturating sigmoidal relationship was observed between the maximum NaCl CT response (CT_max_) and the postnatal age of rat pups. And the apparent affinity of ENaC for Na^+^ declined in rats between 12–14 and 19–23 postnatal days [51].

In 19–23 day old rats, 20 mM 8-CPT-cAMP increased the maximum ENaC-dependent NaCl CT response (*r*_*asm*_) by a factor of 2.18 (Fig 4B). In the Na^+^ channel model [51], *r*_*asm*_ is proportional to the density of functional ENaCs. This suggests that in 19–23 day old rats, 8-CPT-cAMP increases the functional ENaC density by 218%. An increase in *r*_*asm*_ was accompanied by an increase in the voltage sensitivity of ENaC-dependent NaCl CT response (Fig 5). At 0.3 M NaCl, 8-CPT-cAMP (20 mM) treatment increased *κ*_*as*_ by a factor of 11.7 (Table 4). We have previously shown that in adult rats, 8-CPT-cAMP enhanced ENaC-dependent NaCl CT response and the voltage sensitivity of the response at ±60 mV [21]. In adult rats, the control (*κ*_*as0*_) and the post-cAMP (*κ*_*ascAMP*_) mean values at 0.1 M NaCl normalized to 0.3 M NH_4_Cl were 0.0046 and 0.0071 response units/mV, respectively (*κ*_*ascAMP*_/*κ*_*as0*_ = 1.54) [21]. The corresponding values of *κ*_*as0*_ and *κ*_*ascAMP*_ in 19–23 day old rats for 0.1 M NaCl also normalized to 0.3 M NH_4_Cl were 2.4 x 10^−4^ and 5.1 x 10^−3^ response units/mV, respectively (*κ*_*ascAMP*_/*κ*_*as0*_ = 21.3). This large difference in *κ*_*asAMP*_/*κ*_*as0*_ between 19–23 day old rats and adult rats is due to the fact that in developing rats the starting functional ENaC levels are just detectable and cAMP brings them up to adult levels, while in adult rats ENaC levels are closer to their maximum value to start with.

Both in adult rats [21] and in 19–23 day old rats, 8-CPT-cAMP did not have any effect on the Bz-insensitive NaCl CT response (Fig 2F). Therefore, the cAMP-induced increase in NaCl CT response is specifically due to an increase in the ENaC-dependent component of the NaCl CT response. Consistent with this, in rats the amiloride-insensitive NaCl CT response is functional at birth and appears not to vary between 12 and 100 days postnatal [11, 62]. Taken together, the above results indicate that in 19–23 day old rats, an incremental increase in intracellular cAMP mimics the age-dependent increase in the magnitude of NaCl CT response, voltage-sensitivity, and increase in functional ENaCs in the apical membrane of salt sensing FF TRCs.

In our previous studies [63, 64], topical lingual application of 8-CPT-cAMP also enhaned the CT response to strong acids (e.g. HCl) by activating a proton conductance in the apical membrane of sour sensing TRCs. However, no effect of 8-CPT-cAMP was observed on CT responses to sucrose or NaCl + Bz. These studies indicate that 8-CPT-cAMP enhances salty and sour taste responses by activating two different ion channels, ENaC and proton channel(s), respectively, expressed in non-overlapping salty and sour sensing TRCs [20].

In 19–23 day old rats topical lingual application of 20 mM 8-CPT-cAMP enhanced the ENaC-dependent NaCl CT response at 0.1 M NaCl to the level observed in control adult rats (Fig 2F). However, the post-cAMP responses in 19–23 day old rats at 0.3 and 0.5 M NaCl remained significantly lower than in adult rats. The ability of cAMP to enhance ENaC-dependent NaCl CT response in 19–23 day old rats is limited by the expression levels of ENaC subunits in FF TRCs. An increment in ENaC expression and activity is seen in TRCs (Fig 8), reabsorptive epithelia and non-epithelial tissues in a development-dependent manner [26]. Both AVP (Fig 15) and aldosterone [15] have been shown to increase ENaC expression in TRCs and in other epithelial tissues [37–39]. We hypothesize that as TRC ENaC expression increases with postnatal age, 20 mM 8-CPT-cAMP will enhance the ENaC-dependent NaCl CT profile to the level observed in adult rats over the whole range of NaCl concentrations. The presence of amiloride-sensitive whole cell currents in FF TRCs isolated from rats as young as 2 days old has been demonstrated [65]. These results suggest that ENaC is largely in place and functional in rat neonates that nevertheless do not have a detectable ENaC-dependent NaCl CT response until 7–10 days postnatal.

Unlike the mice in which α-ENaC is knocked out globally [29], mice in which α-ENaC has been conditionally knocked out only in TRCs, survive. These mice demonstrate the lack of ENaC-dependent NaCl CT response and preference for appetitive NaCl concentrations [20]. These results indicate in salt sensing FF TRCs both ENaC expression and ENaC trafficking to the apical membrane is necessary for salt taste development.

Placing female pregnant rats on a Na^+^-restricted (0.01% NaCl) diet produces offspring that do not develop functionally active TRC ENaC, provided they are also maintained on the Na^+^-restricted diet into maturity [66]. Normal responses were restored with a latency of about 20 days by providing the Na^+^-restricted rats a one-time access to saline [67]. These studies led to the hypothesis that the ingestion of physiological saline initiated systemic changes leading to hormonal action on the taste system and the activation of TRC ENaC. Rats reared on high (3%) NaCl diet from conception to postnatal day 30 demonstrated greater amiloride-sensitive NaCl responses than rats reared on 1% NaCl diet but gave normal responses when switched to 1% NaCl diet [68, 69]. These results further support the hypothesis that hormonal changes during development regulate TRC ENaC expression and trafficking, and thus the neural and behavioral responses to NaCl.

### Spaciotemporal relationship between cAMP generation and ENaC trafficking

In isolated TRCs, the amiloride-sensitive Na^+^ current is increased by AVP and membrane-permeable cAMP analogues. This effect was predominantly due to an increase in the number of functional apical ENaCs [34]. The maximum increase in the Na^+^-current was observed 15–20 min post-AVP treatment. These results indicate that both AVP-induced increase in intracellular cAMP and ENaC trafficking is a rapid process that occurs in the time frame of minutes. We investigated the spatiotemporal relationship between cAMP generation and ENaC trafficking in HBO cells. HBO cells demonstrated the mRNAs for α-, β-, γ-, and δ-ENaC subunits (Fig 9) and δ- and γ-ENaC proteins (Figs 11–13) [70]. HBO cells treated with 0.1 μM dDAVP or 10 μM forskolin (an activator of adenylyl cyclase) for 10 min increased cAMP (Fig 10), and cAMP, in turn, induced trafficking of δ- and γ-ENaC from cytosolic compartment to the apical compartment in ENaC positive cells (Figs 11–13). Consistent with this, cAMP increased the density of ENaC subunits in the apical membrane of MDCK cells in direct proportion to amiloride-sensitive Na^+^ transport [38]. Cyclic AMP rapidly mobilized wild type human renal ENaC expressed in Xenopus laevis oocytes and increased the membrane density of ENaCs in oocyte membranes but had no effect on the Liddle-mutated hENaC [46]. In our studies intravenous injections of dDAVP induced increased expression and trafficking of γ-ENaC from the cytosolic compartment to the apical membrane of rat FF TRCs (Fig 15). It is important to note that even though 15 day old rats express low levels of TRC V2Rs, repeated injections of high doses of dDAVP (1 nano moles/Kg BW) can induced ENaC expression and trafficking. These results suggest that TRC ENaC is regulated by similar intracellular signaling mechanisms in rodents and humans.

The effect of cAMP on ENaC trafficking is due, at least in part, to inhibition of neural precursor cell expressed developmentally down-regulated protein 4–2 (Nedd4-2)-induced ENaC ubiquitination *via* phosphorylation of Nedd4-2 on Ser^327^, Ser^221^, and Thr^246^. Notably, these are the same residues that are phosphorylated by serum and glucocorticoid-inducible kinase-1 (SGK-1) and protein kinase A (PKA), although the cAMP effect is SGK independent [71, 72]. Nedd4-2 controls ENaC surface expression by catalyzing its ubiquitination, which targets ENaC for degradation [41, 45]. Nedd4-2 binds to the PY motif located in the cytoplasmic C terminus of each ENaC subunit decreasing ENaC surface expression. SGK-1 binds to Nedd4-2 and phosphorylates it which reduces the binding of Nedd4-2 to ENaC [71–75]. Aldosterone acts through the mineralocorticoid receptor to alter the transcription of specific genes, including SGK-1 [42]. Aldosterone has been shown to increase ENaC expression and translocation of ENaC subunits from the cytosolic compartment to the apical membrane of TRCs [15].

In isolated TRCs from adult mice, insulin (5–20 nM) enhanced Na^+^ influx in both patch-clamp and imaging studies. Adult mice injected with insulin showed significant avoidance of NaCl at lower concentrations than the control group [30]. Stimulation of ENaC by insulin involves activation of SGK-1 via phosphoinositide (PI)3-kinase and phosphoinositide-dependent kinase (PDK1) [75]. SGK-1 has been shown to be expressed in vallate and FF taste bud cells [30]. Insulin had no effect on Na^+^ transport in taste cells from SGK^−/−^ mice. In contrast, taste cells from SGK^+/+^ mice evoked greater Na^+^ responses to insulin. Functional ENaC activity was dramatically reduced in SGK^−/−^ taste cells. These studies suggest that SGK is essential to maintain normal ENaC function and that the absence of SGK protein in the null mice severely inhibits insulin's effects in the gustatory system [30]. Thus, it is likely that in addition to cAMP and AVP, aldosterone and insulin also play a role in postnatal development of FF TRC ENaC.

Hormonally induced increase in intracellular cAMP or increasing cell cAMP by direct application of membrane permeable forms of cAMP has been shown to induce a change in cell shape in both epithelial and non-epithelial cells [76–80]. In some studies [77–79], changes in cell shape were observed following incubation of cells with 1 or 2 mM dibutyryl-cAMP or 8-CPT-cAMP. When mesangial cells were treated with 1 mM dibutyryl-cAMP, 85% of cells underwent a shape change by 40 min [76]. Treating cultured rat ovarian granulosa cells with dibutyryl-cAMP resulted in a dose-dependent increase in the percentage of cells that demonstrated morphological change [78]. The above studies suggest that the changes in cell shape induced by an increase in intracellular cAMP is both dose- and time-dependent. In the present study, HBO cells were treated with 10 μM 8-CPT-cAMP for 10 min, a concentration 100 times lower than that used in the above studies. Under these conditions, we expect to observe some changes in the morphology of a subset of HBO cells (Figs 11–13). While there is some change in the cell shape after cAMP or dDAVP treatment, the significant finding is the translocation of γ- and δ-ENaC from the cytosolic compartment to the apical compartment in a subset of HBO cells. It is likely that changes in cell morphology and ENaC subunit translocation from the cytosolic compartment to apical compartment occur in the same time frame. Elevation of intracellular cAMP resulted in a change in cell shape that resembled dome formation in cultured rat glomerular epithelial cells [77]. In Madin-Darby canine kidney epithelial cells (MDCK) an increase in cell cAMP caused an increase in the number and size of hemicysts [80]. This was attributed to a decrease in cell adhesion to the substrate [81]. Cyclic AMP-induced morphological changes are counteracted by the activated RhoA small GTPase and the Rho kinase ROKα [79].

### Physiological implications of cAMP-induced increase in the ENaC-dependent NaCl CT response

In two bottle preference tests, 19–23 day old rats did not discriminate between H_2_O and NaCl concentrations of 0.035, 0.075 and 0.15 M. However, after a single topical lingual application of 20 mM 8-CPT-cAMP for 20 min the 19–23 day old rats demonstrated a clear preference for the above NaCl concentrations (Fig 6). The effects of 8-CPT-cAMP persisted for 5–6 days. 8-CPT-cAMP also produced behavioral changes in adult rats. While in control or 8-CPT-cGMP treated adult rats the maximum intake was observed at 0.15 M NaCl, in 8-CPT-cAMP treated rats the maximum intake was observed at 0.1 M NaCl (Fig 7). This indicates that in adult rats, cAMP-induced increase in TRC ENaC results in shifting the NaCl preference to lower NaCl concentrations. Thus, even in adult rats 8-CPT-cAMP effects could be observed on NaCl behavior over a 7 day period. It is likely that 8-CPT-cAMP is resistant to phosphodiesterases, and therefore, persists intracellularly for extended periods, and thus, maintains the elevated levels of functional apical ENaCs in TRCs. Alternately, during development the cAMP-induced increase in functional apical ENaCs may have a lower rate of degradation due to hormone induced decrease in Nedd4-2 abundance [82].

In summary, the results presented here demonstrate that an incremental increase in FF TRC cAMP mimics the postnatal age-dependent increase in NaCl CT response, increase in functional apical ENaCs, and preference for appetitive NaCl concentrations. AVP binds to V2R coupled to Gα_s_. Gα_s_ stimulates adenylyl cyclase and increases cAMP. During postnatal development an AVP-induced incremental increase in cAMP occurs due to an age-dependent increase in V2Rs in the basolateral membrane of TRCs (Fig 16). This leads to a gradual increase in Gα_s_-induced activation of adenylate cyclase and an incremental increase in TRC cAMP.

## Appendix

### Data analysis

#### Na^+^ concentration and voltage dependence of the CT response

The NaCl CT response is derived from Na^+^ influx via two pharmacologically distinct cation channels: an amiloride- and Bz-sensitive (as) ENaC and an amiloride- and Bz-insensitive (ai) but CPC-sensitive cation channel (Fig 1). The amiloride-sensitive (as) ENaC-dependent NaCl CT response (*r*_*as*_) depends on the NaCl concentration *c*, and the dimensionless transepithelial clamp voltage (*φ*) as follows:

$$r_{aso}=\frac{r_{asm}\;c}{c+K_{as}e^{\delta \varphi }}$$ (1)

Here, *r*_*asm*_ is the maximum response, *K*_*as*_ is the NaCl concentration for which *r*_*as*_ is half maximal when *φ* is zero, referred to as the open-circuit condition. *φ* = F*V*/RT where *V* is the applied clamp voltage in millivolts (referenced to the mucosal side), and RT/F = 25.9 mV and δ is the fraction of the applied voltage dropped across the apical membrane of TRCs expressing ENaC. Similarly, the amiloride- and Bz-insensitive (*ai*) CT response (*r*_*ai*_) is:

$$r_{ai}=\frac{r_{aim}\;c}{c+K_{ai}e^{\lambda \varphi }}$$ (2)

where, the parameters have a similar meaning as in Eq 1. Under open-circuit condition (*φ* = 0) the total NaCl response (*r*_*o*_) is, therefore, the sum:

$$r_{o}=r_{aso}+r_{aio}$$ (3)

#### Voltage effects and response conductance

We have previously shown that in adult rats, 8-CPT-cAMP increased the maximal open-circuit NaCl CT response (*r_asm_*) by increasing the response conductance (*κ*_*as*_). The response conductance is defined as the slope of the CT response curve measured by varying the lingual clamp voltage at a fixed NaCl concentration [21, 55]. The response (*r*) is approximately linear with applied voltage (*V*) (see Fig **5**C). Accordingly, the voltage dependence of the response in Eqs 1 and 2 can be linearized and total response (*r*) appears as follows:

$$r=r_{o}-\kappa V$$ (4)

where, *κ* is the total response conductance:

$$\kappa =\kappa_{as}+\kappa_{ai}$$ (5)

Here,

$$\kappa_{as}=\frac{r_{aso}F\delta }{RT}\left(\frac{K_{as}}{K_{as}+c}\right)$$ (6)

and

$$\kappa_{ai}=\frac{r_{aio}F\lambda }{RT}\left(\frac{K_{ai}}{K_{ai}+c}\right)$$ (7)

From Eqs 6 and 7 we note that the response conductance is proportional to the open-circuit response magnitude for each ion channel type.

#### cAMP concentration dependence of r_asm_ and *K*_*as*_

The 8-CPT-cAMP-induced increase in *r*_*asm*_ was fitted to a sigmoidal saturating function of 8-CPT-cAMP concentration to:

$$r_{asm}=r_{asmo}+\frac{a\;p^{n}}{k^{n}+p^{n}}$$ (8)

and a decrease in *K*_*as*_ was fitted to:

$$K_{as}=\frac{bp^{n}}{q^{n}+p^{n}}$$ (9)

In Eq 8 *r*_*asmc*_ is the value of *r*_*asm*_ when the 8-CPT-cAMP concentration, *p*, is zero, and *a*, *k*, and *n* are positive constants. In Eq 9 *K*_*as*_ is the NaCl concentration (M) at which *r*_*aso*_ becomes half-maximal and *p* is the 8-CPT-cAMP concentration (mM) applied to the rat tongue. The parameter *b* is the *K*_*as*_ value in the untreated tongue for which *p* = 0, *q* is the 8-CPT-cAMP concentration for which *K*_*as*_ reaches half its untreated value (i.e. value at *p* = 0), and *n* is a positive constant.
